# Transcriptome Assembly and Systematic Identification of Novel Cytochrome P450s in *Taxus chinensis*

**DOI:** 10.3389/fpls.2017.01468

**Published:** 2017-08-23

**Authors:** Weifang Liao, Shengying Zhao, Meng Zhang, Kaige Dong, Ying Chen, Chunhua Fu, Longjiang Yu

**Affiliations:** ^1^Department of Biotechnology, Institute of Resource Biology and Biotechnology, College of Life Science and Technology, Huazhong University of Science and Technology Wuhan, China; ^2^Key Laboratory of Molecular Biophysics Ministry of Education, College of Life Science and Technology, Huazhong University of Science and Technology Wuhan, China

**Keywords:** *Taxus chinensis*, cytochrome P450, transcriptome-wide identification, gene expression, Taxol biosynthesis

## Abstract

*Taxus* spp. is a highly valuable medicinal plant with multiple pharmacological effects on various cancers. Cytochrome P450s (CYP450s) play important roles in the biosynthesis of active compounds in *Taxus* spp., such as the famous diterpenoid, Taxol. However, some specific CYP450 enzymes involved in the biosynthesis of Taxol remain unknown, and the systematic identification of CYP450s in *Taxus* has not been reported. In this study, 118 full-length and 175 partial CYP450 genes were identified in *Taxus chinensis* transcriptomes. The 118 full-length genes were divided into 8 clans and 29 families. The CYP71 clan included all A-type genes (52) belonging to 11 families. The other seven clans possessed 18 families containing 66 non-A-type genes. Two new gymnosperm-specific families were discovered, and were named CYP864 and CYP947 respectively. Protein sequence alignments revealed that all of the *T. chinensis* CYP450s hold distinct conserved domains. The expression patterns of all 118 CYP450 genes during the long-time subculture and MeJA elicitation were analyzed. Additionally, the expression levels of 15 novel CYP725 genes in different *Taxus* species were explored. Considering all the evidence, 6 CYP725s were identified to be candidates for Taxol biosynthesis. The cis-regulatory elements involved in the transcriptional regulation were also identified in the promoter regions of CYP725s. This study presents a comprehensive overview of the CYP450 gene family in *T. chinensis* and can provide important insights into the functional gene studies of Taxol biosynthesis.

## Introduction

Taxol (generic name paclitaxel), the main bioactive component of the *Taxus* species, is a highly effective anti-cancer agent widely used in the treatment of various sarcomas, melanomas, and carcinomas (Murphy et al., 1993). However, Taxol availability is restricted due to the insufficient natural supply; thus, Taxol supply and cost remain serious concerns because of its increasing requirements in chemotherapy (Cragg et al., 1993). In the near future, Taxol production and its potential precursors must rely on biological methods, either in *Taxus* tissues or in cell cultures (Ro et al., 2006; Ajikumar et al., 2010; Zhou et al., 2015). Therefore, understanding the biosynthetic pathway of Taxol and the enzymes that catalyze this series of reactions and their underlying molecular mechanism is essential.

The Taxol biosynthetic pathway starts with the cyclization of the universal diterpenoid precursor geranylgeranyl diphosphate to taxa-4(5),11(12)-diene. This taxane core is then decorated with a series of eight Cytochrome P450 (CYP450)-mediated oxidations, three CoA-dependent acylations, and several other transformations that lead to baccatin III, to which the C13-side chain is appended to afford Taxol (Jennewein et al., 2001; Croteau et al., 2006). Thus, CYP450s play a major role in Taxol biosynthesis (Rasool and Mohamed, 2016). Approximately half of the 20 enzymatic steps of the pathway are thought to be catalyzed by CYP450 oxygenases. The proposed order of the oxygenations on the taxane core begins with C5 and C10, then C13 and C9, and later C7 and C2. The final reactions include the epoxidation of the C4, C20-double bond (leading to the oxetane ring) and C1 oxygenation (Wheeler et al., 2001). In the past few years, studies have concentrated on the molecular biochemistry of Taxol biosynthesis (Chau et al., 2004; Wang et al., 2016). However, the CYP450 genes responsible for C1 hydroxylation, oxetane formation, C9 oxidation of the taxane core, and C2′-side-chain hydroxylation in *Taxus* remain unidentified.

CYP450s represent one of the largest gene families and play vital roles in many plant metabolic processes. They catalyze the oxidative modification of various substrates using oxygen and NAD(P)H (Chapple, 1998). Structurally, all plant CYP450s identified so far are membrane-bound enzymes. The vast majority of them are anchored on the endoplasmic reticulum by the hydrophobic signal sequence at the N-terminus (Williams et al., 2000). CYP450 protein sequences contain four unique structures, including the Heme-binding, PERF, and EXXR motifs, located in the K-helix and I-helix. The conserved cystein in the heme-binding motif (F–G-R-C-G) regulates the iron in their heme group. The R amino acid residue of the short string in the PERF motif, together with E and R amino acid residues in the K-helix, forms a salt bridge that is generally considered to be involved in locking the heme pocket into the corresponding position and ensuring the stability of the core structure (Hasemann et al., 1995). CYP450s for all organisms are named and classified by a P450 nomenclature committee (David Nelson: dnelson@uthsc.edu). To differentiate them from other species, plant CYP450s belong to families ranging from CYP71-CYP99, and then from CYP701-CYP999 (Durst and Nelson, 1995; Danielson, 2002). Moreover, CYP450s in plants are classified into two categories: A-type and non-A-type (Paquette et al., 2000). Based on the available sequences, plant CYP450s are further classified into 11 clans (Paquette et al., 2000). A-type CYP450s include only the CYP71 clan, whereas non-A-type CYP450s include 10 clans, namely, CYP51, CYP72, CYP74, CYP85, CYP86, CYP97, CYP710, CYP711, CYP727, and CYP746.

In recent years, the functional characterization of CYP450s involved in terpene biosynthesis has gathered attention. Many studies focusing on the transcriptome-wide identification of CYP450s for terpene biosynthesis have been done. An earlier research performed transcriptomic analyses based on 454 pyrosequencing data from *Panax ginseng* flowers, roots, stems, and leaves, thereby leading to the identitification of 326 potential CYP450s, including CYP716A47, which is related to the ginsenoside biosynthesis (Li C. et al., 2013). In addition, ~300 isotigs similar to CYP450s have been discovered from *Salvia miltiorrhiza* hairy roots using the RNA-Seq technology, of which six were further studied and the CYP76AH1 was functionally confirmed to catalyze the turnover of miltiradiene in tanshinones biosynthesis (Guo et al., 2013). Last year, 70 highly expressed CYP450s in the *Xanthium strumarium* trichomes were studied using an extensive analysis of transcriptomes. Among them, four CYP71 members (CYP71AV14, CYP71BL7, CYP71DD1, and CYP71AX30) were found to be the candidates involved in sesquiterpene lactone biosynthesis (Li et al., 2016). Transcriptome identification of genes for Taxol biosynthesis has been studied, however, no study has demonstrated interest in the CYP450 genes of *Taxus* (Wu et al., 2011; Sun et al., 2013). So, a systematic set of CYP450s and a standard nomenclature could be benefit to the functional identification of CYP450s, which might be involved in the biosynthesis of active ingredients in *Taxus*.

In this study, we established systematic information of CYP450s in *Taxus chinensis* by mining available transcriptome data. We first identified and classified putative full-length CYP450-encoding sequences. Phylogenetic analysis allowed us to identify groups of paralogs for further evaluation. Next, based on the KEGG database, we investigated the potential involvement in various biosynthesis pathways of these CYP450s, with emphasis on taxane biosynthesis. Moreover, the expression profiles *in silico* were characterized, some selected CYP450 genes were confirmed by qRT-PCR. And the expression levels of 15 novel CYP725 genes in different *Taxus* species were also explored. Lastly, the cis-regulatory elements involved in the transcriptional regulation were identified in the promoter regions of CYP725s. Our findings would contribute to advanced research and applications of CYP450 genes in *Taxus*.

## Materials and methods

### *In silico* mining of *T. chinense* CYP450s

All unigenes (containing contigs and singletons) of assembled *T. chinense* transcriptomes (Accession Numbers: SRR1343578, SRR1339474, and GSE28539) were obtained, then, they were clustered using CD-HIT software (Version 4.6), and the sequence identity >90% as the cutoff. The HMM model (PF00067) were retrieved from the Pfam database (http://pfam.sanger.ac.uk). After redundant sequences were removed, the HMMER program was used to identify the CYP450s, with an *e*-value cutoff of 1e-5. The nucleotide sequences of these selected unigenes were subjected to ORF Finder software (http://bioinf.ibun.unal.edu.co/servicios/sms/orf_find.html) for open reading frame (ORF) identification. The full-length CYP450 genes were identified as described by Chen et al. (2014). The search results were further consolidated with previously published genes in Genbank and other existing *Taxus* transcriptomes, including *Taxus* × *media* (SRR534003 and SRR534004), *Taxus mairei* (SRR350719), *Taxus cuspidata* (SRR032523).

### Phylogenetic analysis

For comparison, a collection of CYP450s from *Picea glauca* and corresponding CYP names were obtained from Genbank. Multiple sequence alignment was performed with the ClustalW algorithm-based AlignX module in MEGA 7 software. The phylogenetic tree was constructed by Neighbour-Joining (NJ) method by p-distance in MEGA 7. The significance level for NJ analysis was examined by bootstrapping with 1,000 repeats.

### Classification and characterization of full-length *T. chinense* CYP450s

All full-length CYP450 proteins were classified with reference sequences from an established P450 database (Nelson, 2009). Overall, 40, 55, and 97% sequence identities were used as cutoffs for family, subfamily, and allelic variants, respectively. The names of these CYP450 proteins were assigned by Prof. David Nelson. Theoretical iso-electric points (PI) and molecular weight (kDa) for each full-length CYP450 protein were predicted by ExPASy tool (http://www.expasy.org/tools/). Furthermore, CYP450 motifs were confirmed by Multiple Expectation Maximization for Motif Elicitation (MEME) program (http://alternate.meme-suite.org/). The subcellular locations were conducted using the TargetP1.1 server with specificity >0.95 (http://www.cbs.dtu.dk/services/TargetP/).

### Functional annotation

Functional annotation was performed by searching *T. chinensis* CYP450s against the SWISS-PROT, NR, and NT databases, using BLAST with an *E*-value of 1e-5. The CYP450 genes were also mapped in Kyoto Encyclopedia of Genes and Genomes (KEGG) database to obtain reference metabolic pathways.

### Expression pattern analysis

To analyze the expression levels of CYP450s in different *Taxus* cell lines, we used the reported Solexa sequencing libraries from cell lines CA (subcultured for 10 years) and NA (subcultured for 6 months), and Methyl Jasmonate (MeJA)-mediated *Taxus* cells harvested at 16 h after inoculation (Li et al., 2012; Zhang et al., 2015). Raw expression counts were calculated by FPKM method (reads of fragments per kilobase per million mapped). To identify the differentially expressed genes (DEGs), a greater than two-fold change and a false discovery rate (FDR) ≤ 0.001 were used to determine significant changes in expression. Heatmaps based on raw expression counts were generated with HemI 1.0 (Heatmap Illustrator software, version 1.0). The expression patterns of 17 randomly selected CYP450 genes in CA and NA were validated by qRT-PCR using the same RNA as the Solexa sequencing sample. The RNA were stored at −80°C.

The expression patterns of 15 novel CYP725 genes in different *Taxus* species, including *T. chinensis, T. cuspidate*, and *T. media* were detected using leaves from 5-year-old plants as sample.

Total RNAs was extracted using the RNAprep Pure Plant kit following manufacturer's protocal (TianGen, Beijing). RNA purity and concentration were detected using a NanoDrop_2000 UV-Vis spectrophotometer (Thermo, USA). Approximately 2 μg total RNA was reverse transcribed using the RevertAid™ First Strand cDNA Synthesis Kit (Thermo, USA). cDNA products were diluted 10-fold prior to use for real-time PCR reaction. Gene specific primers were designed by primer premier 5.0 software, and a housekeeping gene (*actin*) was chosen as the internal reference gene. All primer sequences were listed in Table S1. qRT-PCR reactions were performed in 10 μl volume containing 1 μl diluted cDNA, 0.3 μM forward primer, 0.3 μM reverse primer, and 5 μl 2 × SYBR Green PCR Master Mix (Applied Biosystems). The thermal conditions of the qRT-PCR reactions was 95°C for 5 min, and 40 cycles of 95°C for 10 s, 55°C for 10 s, and 72°C for 15 s. Each experiment was performed with three biological and technical replicates. The relative expression levels were calculated using the 2^−ΔΔCT^ method.

### Cis-elements analysis of CYP725 genes

All promoter sequences (2 Kb upstream of initiation codon “ATG”) of CYP725 genes were extracted from the *Taxus* genome (Nystedt et al., 2013) by using GMAP (http://research-pub.gene.com/gmap/). Then, the on-line database PLACE (http://www.dna.affrc.go.jp/PLACE/signalscan.html) was used to identify the cis-acting elements of promoters for each gene (Yan et al., 2017).

## Results

### The construction of *T. chinensis* transcriptomes

To globally identify potential genes in *T. chinensis* transcriptomes were constructed from two *Taxus* cell lines CA and NA (Zhang et al., 2015), and the MeJA-treated *Taxus* cells for 16 h (Tm16) and those of mock-treated cells (Tm0; Li et al., 2012), resulting in 67,147 Unigenes with an average length of 910 bp and N50 of 1,552 bp (Table 1). The GC percentage of the unigenes was 41.37%.

**Table 1:** Statistics of assembly of *T. chinensis* transcriptomes.

**Sample**	**Unigene**	**Total_length (bp)**	**GC (%)**	**N50 (bp)**	**Average (bp)**
CA	39,396	31,110,882	45.07	1428	ND
NA	43,009	31,758,961	45.63	1320	ND
Tm0	39,176	29,459,951	45.69	812	ND
Tm16	38,713	29,896,420	44.96	839	ND
Total	67,147	61,138,078	41.37	1552	910.51

*N50, The largest length L such that 50% of all nucleotides are contained in unigenes of size at least L (de Jong et al., 2001). ND, no data*.

### Identification and classification of *T. chinensis* CYP450s

A total of 118 full-length and 175 partial CYP450 genes were identified in *T. chinensis*. The total number of *T. chinensis* CYP450 genes was 293, which is more than that of *Arabidopsis thaliana* (272). However, without the whole genome sequence, the strict criteria of all *T. chinensis* CYP450 genes were unmet. To further screen for full-length CYP450 genes, we performed BLAST search using standard CYP450 domains against other existing *Taxus* transcriptomes, such as *T. media, T. mairei*, and *T. cuspidata*. No new full-length CYP450 gene was discovered, because their sequencing size was highly limited. Then, the 175 partial CYP450s were assembled using previously known genes in Genbank, and no full-length CYP450s were obtained.

Classification of the 118 full-length CYP450 genes was executed by alignment with CYP450 database (Nelson, 2009) using standard sequence similarity cutoffs, specifically 97, 55, and 40% for allelic, subfamily, and family variants, respectively. Based on these cutoffs, the 118 full-length CYP450s were classified into 8 clans and 29 families and were divided into two categories: A-type (CYP71 clan) and non-A-type (all other clans; Table 2). Among them, only 6 CYP450s have been previously identified, and the other 112 CYP450s were obtained for the first time (Accession Numbers: MF448573-MF448684).

**Table 2:** List of full-length CYP450s of *T. chinensis* identified in this study.

**Name**	**Type**	**Clan**	**Family**	**Subfamily**	**Length**	**Mol. wt (kDa)**	**Theoretical pI**	**TargetP**
TcCYP51G1	non-A	51	CYP51	CYP51G	497	56.56	8.81	S
TcCYP73A170	A	71	CYP73	CYP73A	501	57.50	8.55	S
TcCYP73A171	A	71	CYP73	CYP73A	515	59.07	8.68	S
TcCYP74A73	non-A	74	CYP74	CYP74A	525	58.84	9.1	C
TcCYP74A74	non-A	74	CYP74	CYP74A	474	53.37	6	–
TcCYP74A75	non-A	74	CYP74	CYP74A	475	53.58	5.95	–
TcCYP75A77	A	71	CYP75	CYP75A	504	56.27	9.33	–
TcCYP75B115	A	71	CYP75	CYP75B	516	57.26	6.78	S
TcCYP76AA66	A	71	CYP76	CYP76AA	512	58.05	8.57	S
TcCYP76AA67	A	71	CYP76	CYP76AA	506	57.43	6.58	S
TcCYP76AA68	A	71	CYP76	CYP76AA	505	57.57	9	S
TcCYP76AA69	A	71	CYP76	CYP76AA	507	57.39	8.71	S
TcCYP76AA70	A	71	CYP76	CYP76AA	513	58.57	8.77	S
TcCYP76AA71	A	71	CYP76	CYP76AA	523	59.13	7.58	–
TcCYP76AA72	A	71	CYP76	CYP76AA	505	57.68	8.73	S
TcCYP76AA73	A	71	CYP76	CYP76AA	523	59.71	9.03	–
TcCYP76Z4	A	71	CYP76	CYP76Z	511	57.63	9.08	–
TcCYP77F31	A	71	CYP77	CYP77F	508	57.18	9.03	S
TcCYP77F32	A	71	CYP77	CYP77F	503	57.05	8.54	S
TcCYP77F33	A	71	CYP77	CYP77F	525	59.16	9.06	–
TcCYP77F34	A	71	CYP77	CYP77F	461	51.55	8.62	–
TcCYP77F35	A	71	CYP77	CYP77F	503	56.85	8.77	S
TcCYP78A233	A	71	CYP78	CYP78A	554	61.89	8.66	–
TcCYP78A234	A	71	CYP78	CYP78A	548	61.33	8.93	–
TcCYP86A149	non-A	86	CYP86	CYP86A	523	59.27	8.93	S
TcCYP86J5	non-A	86	CYP86	CYP86J	540	61.85	8.58	–
TcCYP86P14	non-A	86	CYP86	CYP86P	491	55.59	9.32	S
TcCYP90A54	non-A	85	CYP90	CYP90A	487	55.26	8.64	S
TcCYP94D79	non-A	86	CYP94	CYP94D	519	59.13	8.87	S
TcCYP94D80	non-A	86	CYP94	CYP94D	522	59.50	8.85	S
TcCYP94D81	non-A	86	CYP94	CYP94D	517	59.23	7.59	S
TcCYP94D82	non-A	86	CYP94	CYP94D	514	58.80	8.43	S
TcCYP94D83	non-A	86	CYP94	CYP94D	515	58.21	8.59	S
TcCYP94P4	non-A	86	CYP94	CYP94P	501	57.22	7.1	S
TcCYP94P5	non-A	86	CYP94	CYP94P	510	57.80	9.31	S
TcCYP94P6	non-A	86	CYP94	CYP94P	530	60.00	6.63	S
TcCYP94P7	non-A	86	CYP94	CYP94P	518	58.50	9.11	S
TcCYP97A57	non-A	97	CYP97	CYP97A	673	75.86	8.96	C
TcCYP97B52	non-A	97	CYP97	CYP97B	584	65.81	8.26	C
TcCYP701A59	A	71	CYP701	CYP701A	507	58.32	6.97	S
TcCYP703D4	A	71	CYP703	CYP703D	524	59.78	9.23	S
TcCYP704C10	non-A	86	CYP704	CYP704C	510	59.12	7.28	S
TcCYP710A78	non-A	710	CYP710	CYP710A	499	57.33	8.05	S
TcCYP715C54	non-A	72	CYP715	CYP715C	532	60.09	9.01	–
TcCYP715D11	non-A	72	CYP715	CYP715D	525	59.67	8.65	S
TcCYP716B29	non-A	85	CYP716	CYP716B	484	54.52	8.46	S
TcCYP716B30	non-A	85	CYP716	CYP716B	475	53.70	8.54	S
TcCYP716B31	non-A	85	CYP716	CYP716B	475	53.56	8.64	S
TcCYP718B1	non-A	85	CYP718	CYP718B	482	55.20	8.61	S
TcCYP720B23	non-A	85	CYP720	CYP720B	485	56.18	8.48	S
TcCYP720B24	non-A	85	CYP720	CYP720B	485	55.99	8.83	S
TcCYP720B25	non-A	85	CYP720	CYP720B	486	56.12	8.67	S
TcCYP720B26	non-A	85	CYP720	CYP720B	486	55.87	8.64	S
TcCYP725A1	non-A	85	CYP725	CYP725A	497	56.55	9.14	–
TcCYP725A2	non-A	85	CYP725	CYP725A	485	54.49	9.04	S
TcCYP725A3	non-A	85	CYP725	CYP725A	497	55.86	8.77	S
TcCYP725A4	non-A	85	CYP725	CYP725A	502	56.85	8.13	–
TcCYP725A5	non-A	85	CYP725	CYP725A	493	55.32	9.41	S
TcCYP725A6	non-A	85	CYP725	CYP725A	495	55.53	9.35	S
TcCYP725A9	non-A	85	CYP725	CYP725A	493	55.35	8.68	S
TcCYP725A10	non-A	85	CYP725	CYP725A	484	54.56	9.1	S
TcCYP725A11	non-A	85	CYP725	CYP725A	509	58.07	9.48	–
TcCYP725A12	non-A	85	CYP725	CYP725A	479	54.11	9.01	–
TcCYP725A13	non-A	85	CYP725	CYP725A	484	54.50	8.98	S
TcCYP725A14	non-A	85	CYP725	CYP725A	484	55.03	8.85	S
TcCYP725A15	non-A	85	CYP725	CYP725A	479	54.20	9.21	–
TcCYP725A16	non-A	85	CYP725	CYP725A	478	54.12	9.17	–
TcCYP725A17	non-A	85	CYP725	CYP725A	484	55.09	9.03	S
TcCYP725A18	non-A	85	CYP725	CYP725A	498	56.29	9.11	S
TcCYP725A19	non-A	85	CYP725	CYP725A	497	55.82	8.89	S
TcCYP725A20	non-A	85	CYP725	CYP725A	503	56.79	8.91	–
TcCYP725A21	non-A	85	CYP725	CYP725A	509	58.25	9.4	–
TcCYP725A22	non-A	85	CYP725	CYP725A	499	57.24	8.77	–
TcCYP725A23	non-A	85	CYP725	CYP725A	489	55.66	9.16	S
TcCYP728Q11	non-A	85	CYP728	CYP728Q	490	56.10	9.28	–
TcCYP728Q12	non-A	85	CYP728	CYP728Q	483	54.47	8.79	S
TcCYP728Q13	non-A	85	CYP728	CYP728Q	479	54.76	9.02	S
TcCYP728S2	non-A	85	CYP728	CYP728S	494	55.70	9.06	–
TcCYP728S3	non-A	85	CYP728	CYP728S	482	54.23	8.98	S
TcCYP729B25	non-A	85	CYP729	CYP729B	482	55.11	9.1	S
TcCYP736C8	A	71	CYP736	CYP736C	509	57.51	6.48	S
TcCYP736E20	A	71	CYP736	CYP736E	514	58.03	6.72	C
TcCYP736E21	A	71	CYP736	CYP736E	503	56.85	6.82	S
TcCYP736E22	A	71	CYP736	CYP736E	503	56.83	6.79	S
TcCYP736E23	A	71	CYP736	CYP736E	502	56.53	6.77	C
TcCYP736E24	A	71	CYP736	CYP736E	514	58.18	6.56	S
TcCYP750B2	A	71	CYP750	CYP750B	507	57.00	6.93	S
TcCYP750C16	A	71	CYP750	CYP750C	517	59.02	6.23	–
TcCYP750C17	A	71	CYP750	CYP750C	525	59.76	7.11	S
TcCYP750C18	A	71	CYP750	CYP750C	520	58.93	7.62	S
TcCYP750C19	A	71	CYP750	CYP750C	526	60.31	6.5	S
TcCYP750C20	A	71	CYP750	CYP750C	526	60.10	6.39	S
TcCYP750C21	A	71	CYP750	CYP750C	512	57.97	6.74	–
TcCYP750C22	A	71	CYP750	CYP750C	521	59.26	7.23	S
TcCYP750C23	A	71	CYP750	CYP750C	518	58.57	8.77	–
TcCYP750C24	A	71	CYP750	CYP750C	522	59.54	7.21	–
TcCYP750C25	A	71	CYP750	CYP750C	509	57.13	7.63	S
TcCYP750C8	A	71	CYP750	CYP750C	508	57.16	8.03	–
TcCYP750C3	A	71	CYP750	CYP750C	531	59.70	8.4	C
TcCYP750C26	A	71	CYP750	CYP750C	512	57.45	6.09	–
TcCYP750C27	A	71	CYP750	CYP750C	509	57.09	8.38	S
TcCYP750C28	A	71	CYP750	CYP750C	513	57.94	7.63	S
TcCYP750C29	A	71	CYP750	CYP750C	512	57.77	7	S
TcCYP750C30	A	71	CYP750	CYP750C	484	54.39	8.37	S
TcCYP782B7	A	71	CYP782	CYP782B	508	58.10	6.96	S
TcCYP864B7	non-A	72	CYP864	CYP864B	519	58.62	8.06	S
TcCYP866A17	non-A	72	CYP866	CYP866A	520	58.89	9.36	S
TcCYP866A18	non-A	72	CYP866	CYP866A	515	58.36	9.24	S
TcCYP866A19	non-A	72	CYP866	CYP866A	515	58.26	9.38	S
TcCYP866A20	non-A	72	CYP866	CYP866A	515	58.45	9.18	S
TcCYP866B7	non-A	72	CYP866	CYP866B	530	59.96	9.44	S
TcCYP866B10	non-A	72	CYP866	CYP866B	516	58.46	9.29	S
TcCYP867B5	A	71	CYP867	CYP867B	509	57.32	8.98	C
TcCYP867E3	A	71	CYP867	CYP867E	507	57.31	9.16	–
TcCYP867F22	A	71	CYP867	CYP867F	526	59.38	6.39	–
TcCYP867F23	A	71	CYP867	CYP867F	532	60.10	7.06	S
TcCYP867G20	A	71	CYP867	CYP867G	512	58.19	7.73	S
TcCYP947A88	non-A	85	CYP947	CYP947A	496	56.18	9.09	S

*T. chinensis* CYP450s were further compared with three other typical plant species, such as angiosperms *A. thaliana* and *Medicago sativa* and the gymnosperm *P. glauca* (Tables 3, 4). The results showed that CYP750 was the largest A-type family in *T. chinensis* but absent in *A. thaliana* and *M. sativa*. Conversely, CYP71, CYP79, CYP81, CYP82, CYP83, and CYP89 families were found in *A. thaliana* and *M. sativa*, but not in *T. chinensis* and *P. glauca*. For the non-A-type CYP450s, the CYP725, and CYP866 families were found in *T. chinensis* and *P. glauca*, but absent in *A. thaliana* and *M. sativa*. CYP72, CYP87, CYP96, CYP714, CYP721, and CYP722 families were only found in *A. thaliana* and *M. sativa*. Moreover, two new gymnosperm-specific families, named CYP864 and CYP947, were discovered in *T. chinensis*. The above results showed that distinct genetic differences exist between the gymnosperm and angiosperm, and *P. glauca* is a good reference for *T. chinensis* in comparative studies of CYP450 genes.

**Table 3:** Comparison of A-type CYP450 families among *A. thaliana* (At), *M. sativas* (Ms), *P.glauca* (Pg) and *T.chinensis* (Tc).

**Family**	**At**	**Ms**	**Pg**	**Tc**
CYP71	52	37	0	0
CYP79	7	3	0	0
CYP81	18	5	0	0
CYP82	5	10	0	0
CYP83	2	9	0	0
CYP89	7	9	0	0
CYP750	0	0	6	18
CYP867	0	0	1	5
CYP73	1	1	1	2
CYP75	1	0	6	2
CYP76	8	6	5	9
CYP77	5	2	1	5
CYP78	6	1	6	2
CYP92	0	1	2	0
CYP98	3	1	1	0
CYP701	1	1	1	1
CYP703	1	1	0	1
CYP705	26	0	0	0
CYP736	0	1	17	6
CYP782	0	0	0	1

**Table 4:** Comparison of non-A-type CYP450 families among *A. thaliana* (At), *M. sativas* (Ms), *P. glauca* (Pg), and *T. chinensis* (Tc).

**Family**	**At**	**Ms**	**Pg**	**Tc**
CYP72	9	7	0	0
CYP87	1	2	0	0
CYP96	13	5	0	0
CYP714	2	3	0	0
CYP721	1	1	0	0
CYP722	1	1	0	0
CYP725	0	0	1	21
CYP866	0	0	3	6
CYP51	1	1	1	1
CYP74	2	4	5	3
CYP85	2	1	1	0
CYP86	11	3	18	3
CYP88	2	3	1	0
CYP90	4	4	3	1
CYP94	6	4	1	9
CYP97	3	4	4	2
CYP704	3	14	4	1
CYP707	4	3	2	0
CYP710	4	1	1	1
CYP711	1	2	1	0
CYP715	1	1	0	2
CYP716	2	3	3	3
CYP718	1	0	0	1
CYP720	1	1	5	4
CYP724	1	0	1	0
CYP727	0	0	1	0
CYP728	0	0	0	5
CYP729	0	1	0	1
CYP864	0	0	0	1
CYP947	0	0	0	1

### Phylogenetic analysis

**Figure 1:**
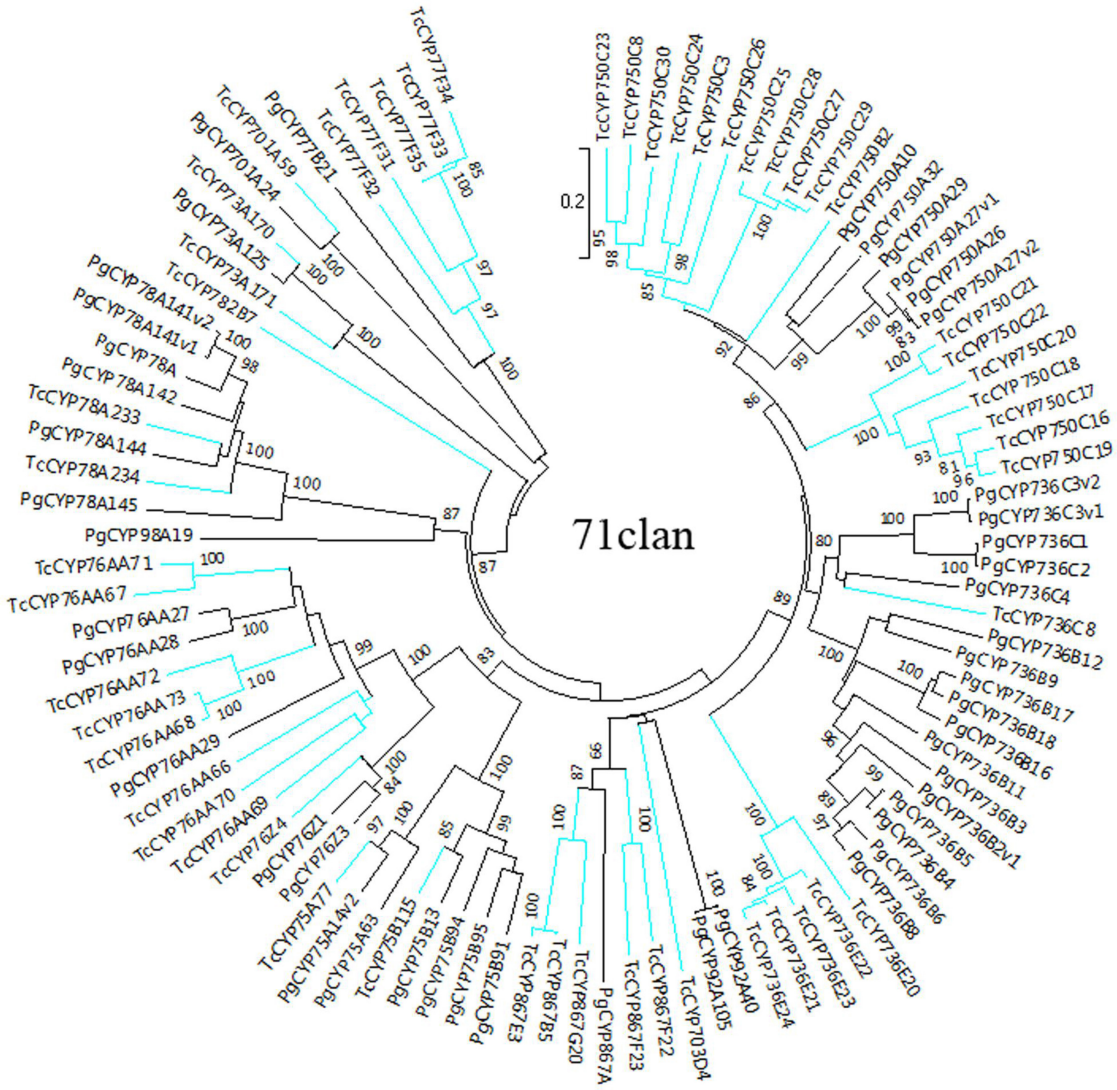
Phylogenetic tree of A-type CYP450 proteins from *T. chinensis* (Tc) and *P. glauca* (Pg). The spokes corresponding to Tc and Pg CYP450s are shown in blue and black, respectively.

**Figure 2:**
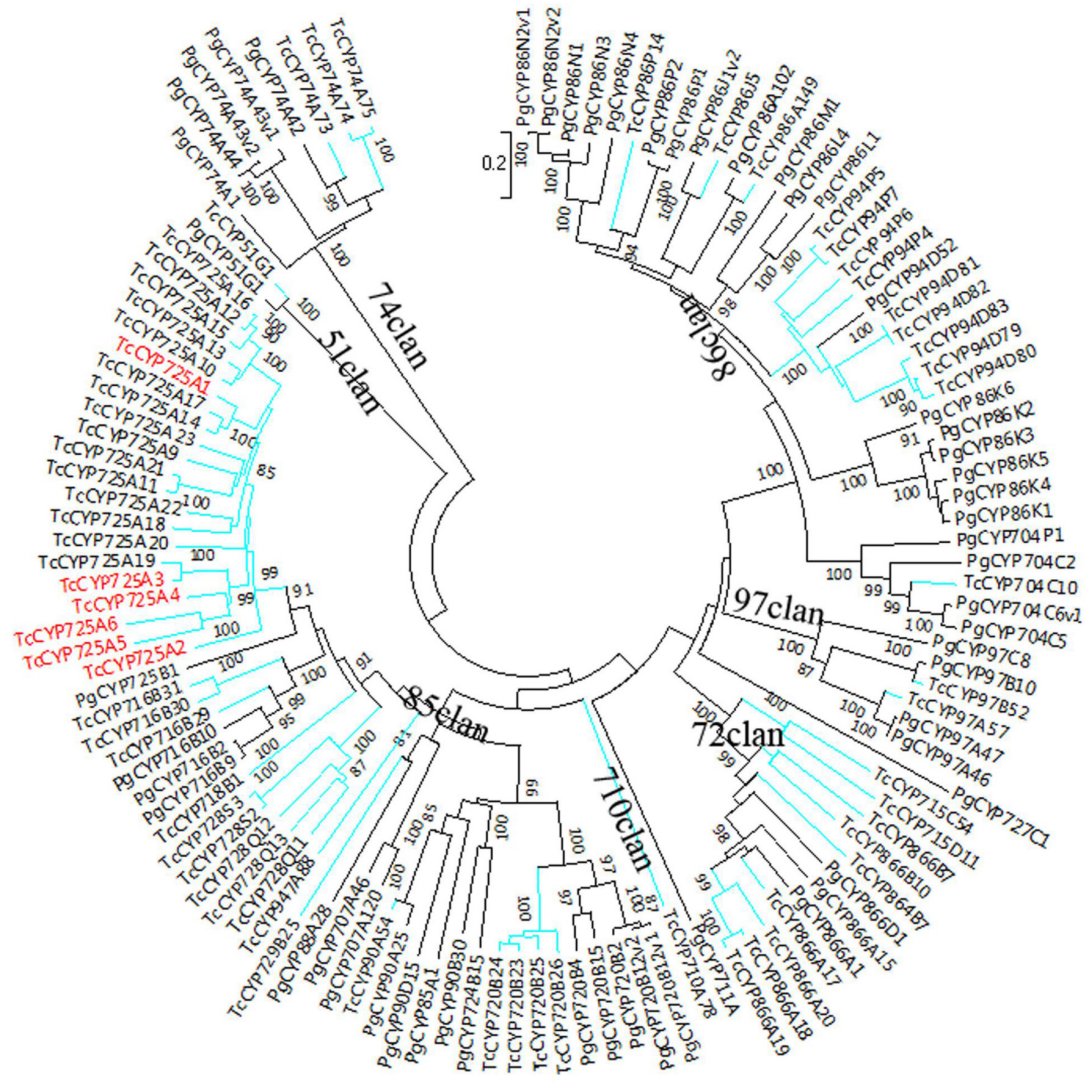
Phylogenetic tree of non-A-type CYP450 proteins from *T. chinensis* (Tc) and *P. glauca* (Pg). The spokes corresponding to Tc and Pg CYP450s are shown in blue and black, respectively. The genes marked in red are described in the text.

The sequences of *P. glauca* CYP450 proteins and the 118 full-length *T. chinensis* CYP450 proteins were used to construct NJ phylogenetic trees for A-type (Figure 1) and non-A-type (Figure 2) CYP450s, separately, using the MEGA7 package. The results showed that *P. glauca* subfamilies are grouped with *T. chinensis* CYP450s. Based on phylogenetic trees, 44.1% (52 genes) of the 118 full-length CYP450s are A-type and belong to 11 families. The remaining 55.9% (66 genes) CYP450s are non-A-type and are distributed to 18 families and 7 clans. The A-type CYP450s (71 clan) have been identified to be related to the biosynthesis of secondary compounds. The Figure 2 showed that non-A-type CYP450s include a more diverse group of genes belonging to the remaining 7 clans. These genes involved in the metabolic pathways of primary products (such as carotenoid, oxylipin, etc.), plant hormone and secondary products. 5 CYP450s have been previously identified to be involved in Taxol biosynthesis, such as TcCYP725A1 (taxane 5-alpha-taxadienol-10-beta-hydroxylase, T10βH), TcCYP725A2 (taxane 13-alpha-hydroxylase, T13αH), TcCYP725A4 (taxadiene 5-alpha-hydroxylase, T5αH), TcCYP725A5 (taxoid 7-beta-hydroxylase, T7βH), and TcCYP725A6 (taxoid 2-alpha-hydroxylase, T2αH). In addition, TcCYP725A3, which is highly homologous with taxane 14β-hydroxylase (T14βH) in *T. cuspidata*. It is suggested that the CYP725A subfamily underwent independent evolution to carry its unique function.

### Physicochemical and structural analyses of *T. chinensis* CYP450s

The physicochemical parameters of each CYP450 gene were calculated using ExPASy. Most of the members had relative molecular weights close to 55 kDa. Approximately four-fifths of the CYP450 proteins had relatively high isoelectric points (pI > 7); the remaining proteins, particularly those in CYP736 family, had pI < 7. TargetP was used to predict the localizations of 118 *T. chinensis* CYP450 proteins, most of them were predicted to be anchored on the endoplasmic reticulum. Hitherto, no plant CYP450s have been found to be located in the mitochondria.

All typical conserved structures of CYP450 proteins were present in non-A-type *T. chinensis* CYP450s (Figure S1), including the cysteine heme-iron ligand signature motif (with PFG element), the PERF motif, K-helix region, and I-helix region. Interestingly, the conserved I-helix region did not exist in A-type *T. chinensis* CYP450s. For the heme-binding motifs, the A-type CYP450s displayed the signature “PFGxGRRxCxG,” whereas “xFxxGxRxCxG” was found in non-A-type CYP450s. Consistent with previous studies, PERF motifs of A-type and non-A-type CYP450s were different. In *T. chinensis*, PERF motifs were “PERF” for A-type and “FxPx” for non-A-type. Moreover, the EXXR motif of A-type was consistent with non-A-type CYP450 proteins. These elements ensure structural stability and flexibility, thereby enabling proteins to bind to appropriate substrates.

### Annotation of *T. chinensis* CYP450s

KEGG pathway-based analysis was performed to further understand the functions of the CYP450 genes. In total, the 118 full-length CYP450s were assigned to 16 KEGG pathways (Figure S2). Significantly, only 2 CYP450s were mapped to the diterpenoid biosynthesis pathway, including TcCYP729B25 and TcCYP701A59. CYP701 is related to the biosynthesis of diterpenoid acids, gibberellins.

Information on the specific metabolic pathways in gymnosperms was highly limited, resulting the CYP725s that are related to diterpenoid Taxol biosynthesis were mapped to the carotenoid biosynthesis pathway. The most represented CYP725 family in *Taxus* (Table 4) plays an important role in the biosynthesis of the diterpenoid anti-cancer drug, Taxol. Moreover, the acquired T2αH, T5αH, T7βH, T10βH, T13αH, and T14βH have high sequence similarity with each other. With an amino acid sequence similarity higher than 70%, the taxoid CYP450 monooxygenases are more conserved than any other known plant of the CYP450 type. Taxoid hydroxylases, with their unique structures and substrate selectivities, form an especially cohesive group. The novel CYP725 proteins identified in this study may be related to Taxol biosynthesis. However, the functional importance of these proteins remains to be determined.

### Expression profile analysis of CYP450s

Illumina transcriptome sequencing technology was used to analyze the gene expressions partners of all 118 full-length CYP450s. These data sets were generated from total RNAs isolated from two *Taxus* cell lines, CA and NA, and MeJA-mediated *Taxus* cells harvested 16 h after inoculation (Li et al., 2012; Zhang et al., 2015).

**Figure 3:**
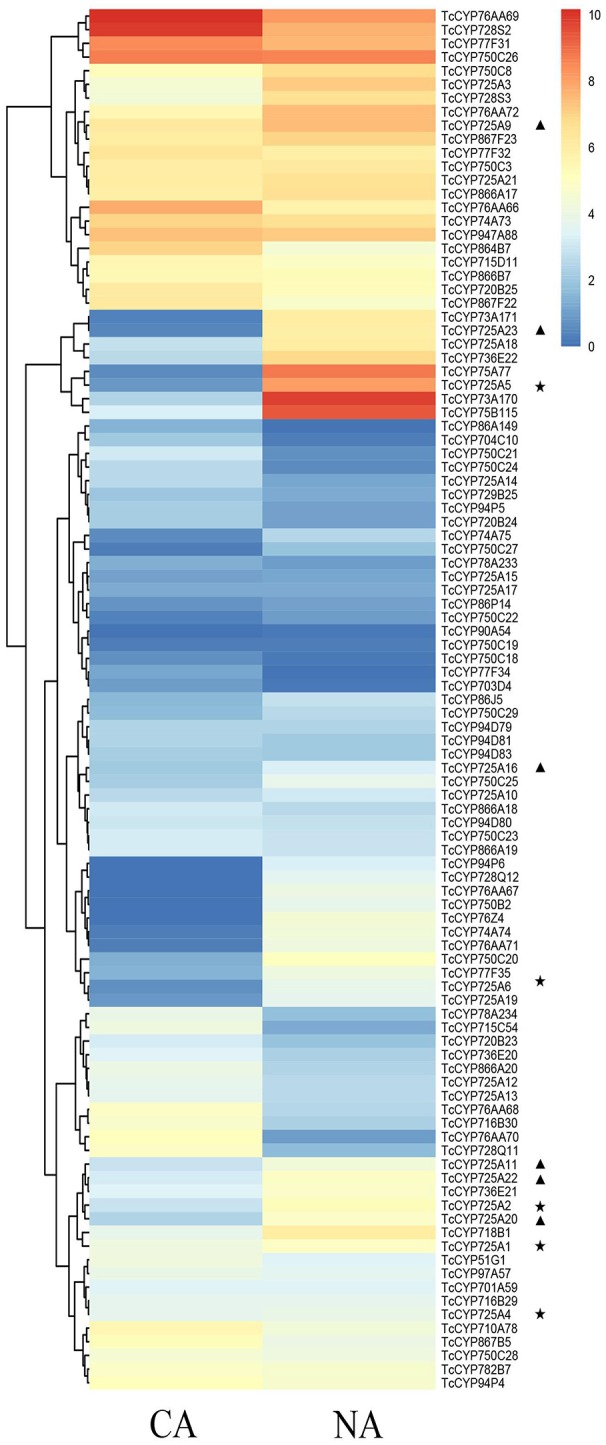
Expression pattern of *T. chinensis* CYP450s in CA and NA cell lines according to the analysis of RNA-Seq dataset. The color scale shows the expression quantity (red: high expression; blue: low expression). Heat map was created using HemI. Heatmap Illustrator v1.0. “⋆” indicates the known taxoid hydroxylase genes, and “▴” indicates the candidate genes.

**Figure 4:**
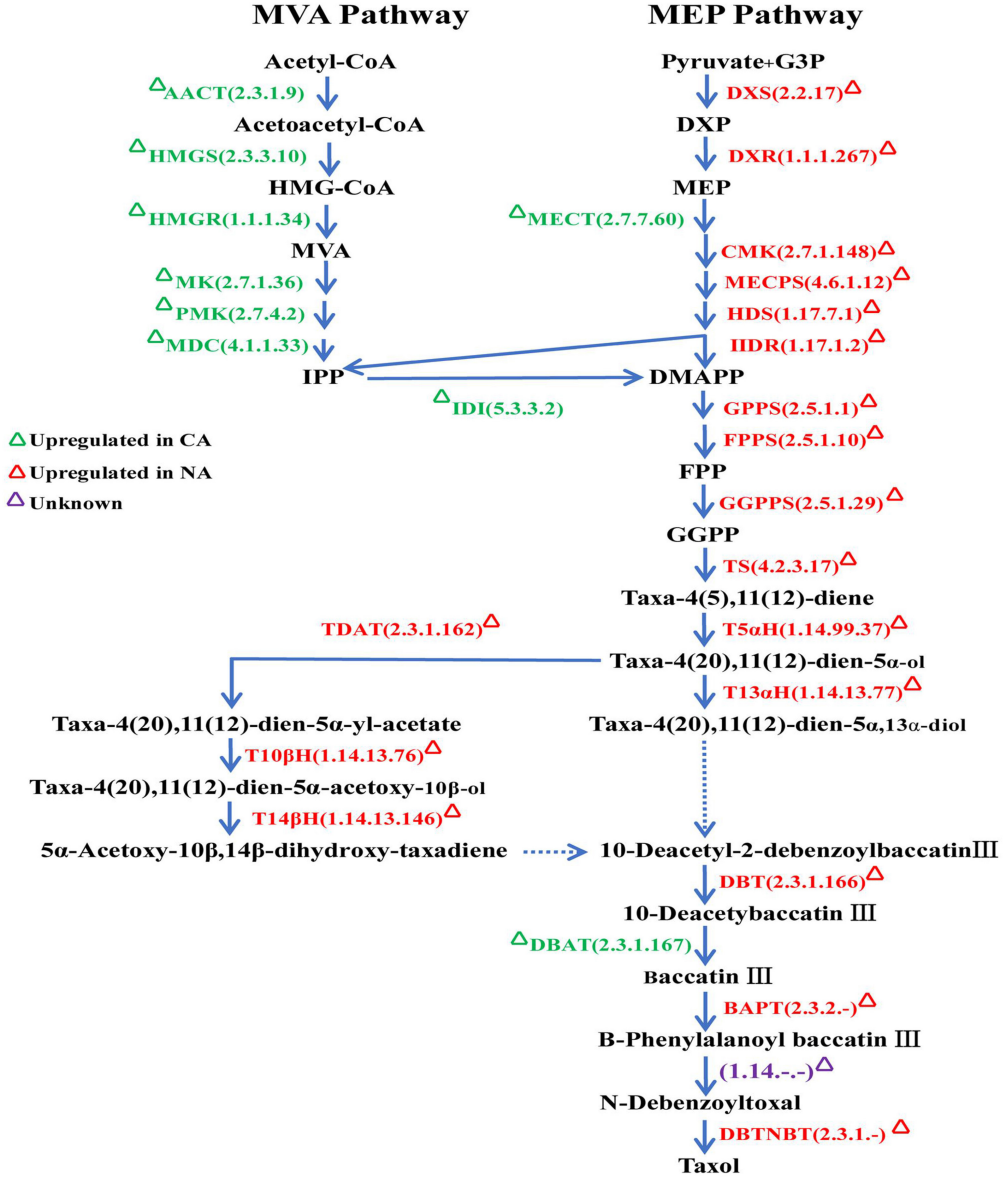
Schematic representation of the taxol biosynthetic pathway. Enzymes were marked according to their specific expression in CA and NA. AACT, acetyl-CoA C-acetyltransferase; HMGS, hydroxymethylglutaryl-CoA synthase; HMGR, hydroxymethylglutaryl-CoA reductase; MK, mevalonate kinase; PMK, phosphomevalonate kinase; MDC, diphosphomevalonate decarboxylase; DXS, 1-deoxy-D-xylulose-5-phosphate synthase; DXR, 1-deoxy-D-xylulose-5-phosphate reductoisomerase; MECT, 2-C-methyl-D-erythritol 4-phosphate cytidylyltransferase; CMK, 4-diphosphocytidyl-2-C-methyl-D-erythritol kinase; MECPS, 2-C-methyl-D-erythritol 2,4-cyclodiphosphate synthase; HDS, (E)-4-hydroxy-3-methylbut-2-enyl-diphosphate synthase; HDR, 4-hydroxy-3-methylbut-2-en-1-yl diphosphate reductase; IDI, isopentenyl-diphosphate Delta-isomerase; GPPS, geranyl diphosphate synthase; FPPS, farnesyl diphosphate synthase; GGPPS, geranylgeranyl diphosphate synthase; TS, taxadiene synthase; T5αH (CYP725A4), taxadiene 5alpha-hydroxylase; T13αH (CYP725A2), taxane 13-alpha-hydroxylase; TDAT, taxadien-5-alpha-ol O-acetyltransferas; T10βH (CYP725A1), taxane 10-beta-hydroxylase; T14βH (CYP725A3), taxoid 14beta-hydroxylase; DBT, 2-alpha-hydroxytaxane 2-O-benzoyltransferase; DBAT, 10-deacetylbaccatinIII 10-O-acetyltransferase; BAPT, Baccatin III amino phenylpropanoyl-13-O-transferase; DBTNBT, 3′-*N*-debenzoyl-2′-deoxytaxol *N*-benzoyl transferase.

From these Illumina data, all 118 full-length CYP450 genes expressed in different *Taxus* cell lines, CA and NA, were hierarchically clustered using HemI Heatmap Illustrator v1.0 software (Figure 3, Table S2). According to previous research (Song et al., 2014), contents of secondary metabolites in NA were significantly higher than in CA. The amount of Taxol was 1.88 times higher than that in CA. The downregulation of secondary metabolites in CA may be due to the decreased activity of specific enzymes, including CYP450 monooxygenases. Figure 4 showed highly different expression profiles in NA and CA. TcCYP73A171, TcCYP74A74, TcCYP75A77, TcCYP75B115, TcCYP76AA71, TcCYP76Z4, TcCYP728Q12, TcCYP736E22, and TcCYP750C20 were lowly expressed in CA, but were highly expressed in NA. Moreover, TcCYP76AA67, TcCYP90A54, and TcCYP750B2 members were not detected in CA. CYP450 oxygenases that potentially related to the Taxol biosynthesis were mainly analyzed. The identified transcripts involved in the Taxol biosynthetic pathway and their specific expression levels in CA and NA were shown in Figure 4. Most enzymes of the native methylerythritol phosphate (MEP) pathway were highly expressed in NA (Table S3). Co-expression with a Taxol biosynthesis marker gene, taxadiene synthase (TS), the acquired taxoid hydroxylases were also highly expressed in NA. The expression levels of T2αH and T7βH greatly increased more than 10-fold, T10βH and T13αH increased two-fold, and T5αH increased slightly. In this study, 10 novel CYP725A genes (TcCYP725A9, TcCYP725A10, TcCYP725A11, TcCYP725A16, TcCYP725A18, TcCYP725A19, TcCYP725A20, TcCYP725A21, TcCYP725A22, and TcCYP725A23) showed higher expression levels in NA.

**Figure 5:**
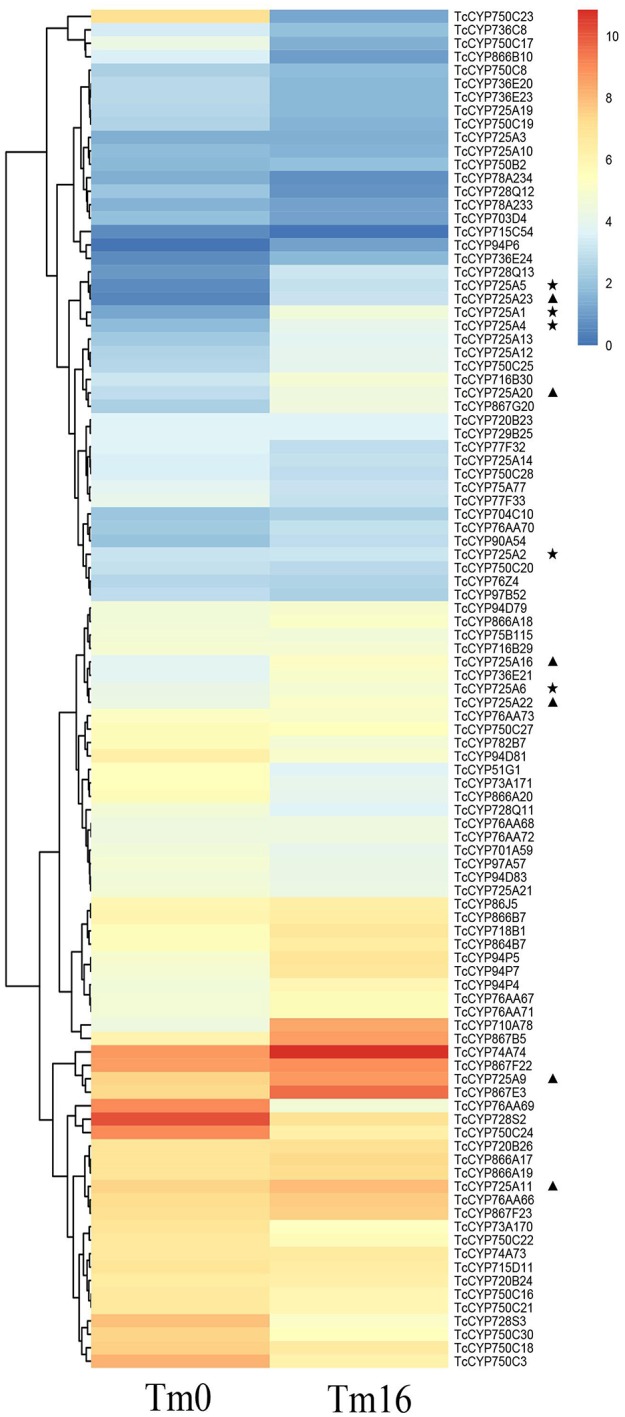
Expression pattern of *T. chinensis* CYP450s under MeJA elicitationin according to the analysis of RNA-Seq dataset. The color scale shows the expression quantity (red, high expression; blue, low expression). Heat map was created using HemI. Heatmap Illustrator v1.0. “⋆” indicates the known taxoid hydroxylase genes, and “▴” indicates the candidate genes.

Previous research confirmed that taxane biosynthesis is regulated by MeJA elicitation in *T. chinensis* cells (Li et al., 2012). Transcriptome profiles of *T. chinensis* cells at 16 h (Tm16) after MeJA treatment and those of mock-treated cells (Tm0) showed that the mRNA levels of most defined hydroxylase genes for taxol biosynthesis increased at 16 h after MeJA elicitation; moreover, genes corresponding to T5αH, T7βH, and T10βH were significantly up-regulated, and genes corresponding to T2αH and T13αH were slightly up-regulated (Table S3). For the 15 novel CYP725 genes, TcCYP725A9, TcCYP725A11, TcCYP725A12, TcCYP725A13, TcCYP725A16, TcCYP725A20, TcCYP725A22, and TcCYP725A23 were up-regulated at 16 h after MeJA elicitation (Figure 5, Table S4).

**Figure 6:**
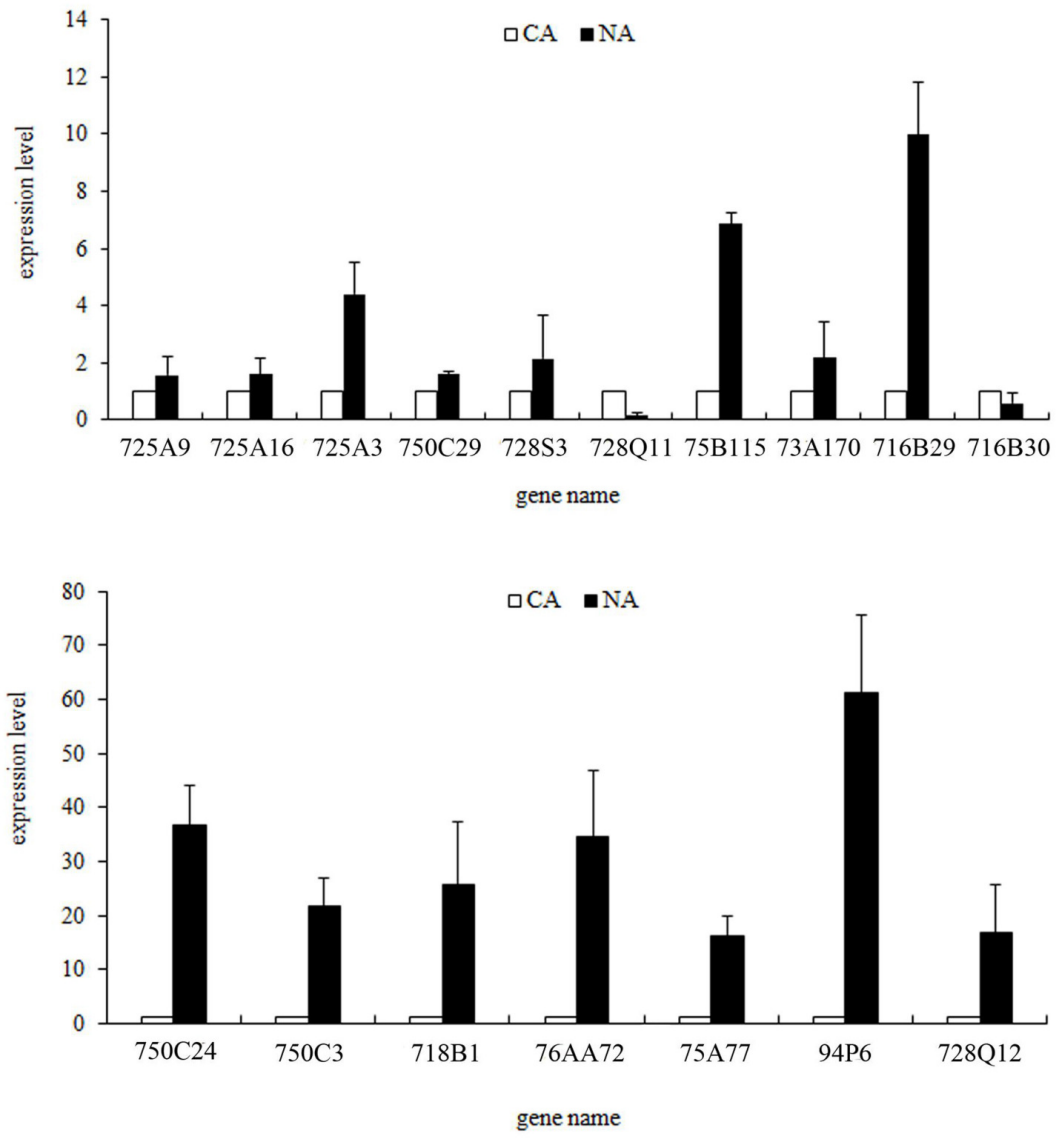
qRT-PCR confirmation of the expression profiles of some randomly selected CYP450 genes. Fold changes of transcript levels in CA and NA are shown. Error bars indicate the mean (SEM).

To verify expression profiles obtained from Illumina sequencing, we performed qRT-PCR on 17 randomly selected CYP450 genes (Figure 6, Table S5). Consistent with the Illumina data, most genes showed strong expression levels in NA. The expression fold changes of some genes, such as TcCYP73A170, TcCYP716B29, TcCYP750C3, and TcCYP716B were higher than RNA-seq results. qRT-PCR results validated that the RNA-seq data is reliable.

**Figure 7:**
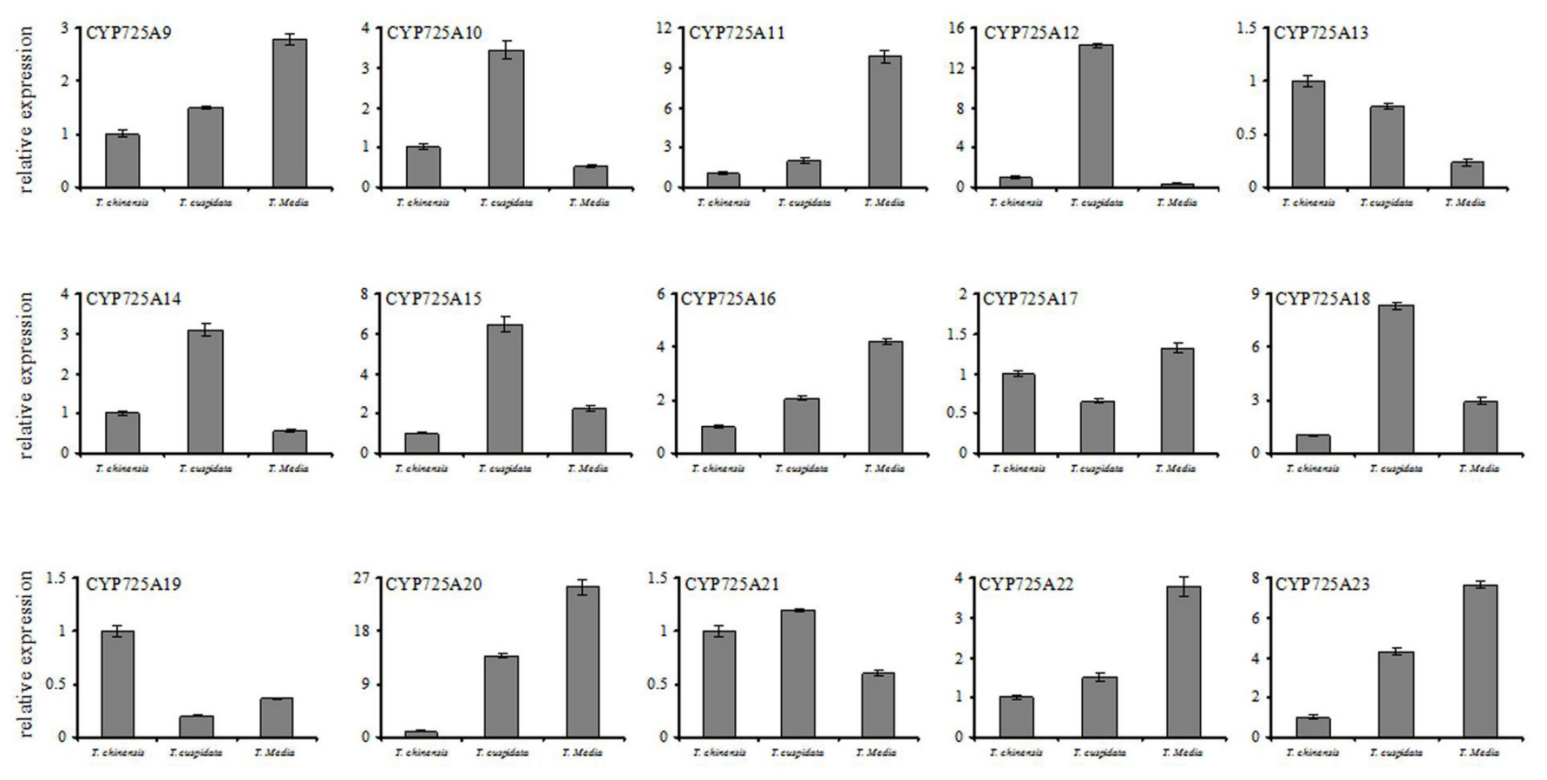
Comparisons of the expression profiles of 15 novel CYP725 genes in different 5-year-old *Taxus* species, including *T. chinensis, T. cuspidate*, and *T. Media*. The bars represent the standard deviation (*n* = 3).

The different expression levels of 15 novel CYP725 genes among three different species such as *T. chinensis, T. cuspidate*, and *T. media* were also investigated (Figure 7). The qRT-PCR profiles showed that CYP725 genes had different expression profiles in different *Taxus* species. All genes but CYP725A13 and CYP725A19 showed a low expression level in *T. chinensis. Six* genes (TcCYP725A9, TcCYP725A11, TcCYP725A16, TcCYP725A20, TcCYP725A22, and TcCYP725A23) showed the highest expression level in *T. media*, followed by *T. cuspidata*, and little amount in *T. chinensis*. The taxol content in *T. media* (0.0186%) was higher than *T. cuspidata* (0.0138%) and *T. chinensis* (0.0109%). The expression rule of these six genes were coincident with Taxol content.

### Identification of candidates CYP450s involved in taxol biosynthesis

CYP450s that were potentially related to Taxol biosynthesis were further identified based on the three following criteria: (1) belonging to CYP725 family. To date, the CYP450 enzymes identified in Taxol biosynthesis are primarily CYP725s, (2) expression level was corresponding with the known Taxol biosynthesis hydroxylases CYP725A1, CYP725A2, CYP725A4, CYP725A5, and CYP725A6, (3) expression profile was consistent with Taxol content. Among the 118 full-length CYP450s, 15 novel genes belong to the CYP725 family (from CYP725A9 to CYP725A23). More importantly, TcCYP725A9, TcCYP725A11, TcCYP725A16, TcCYP725A20, TcCYP725A22, and TcCYP725A23 were highly expressed in NA and up-regulated after MeJA elicitation. The expression pattern of these six CYP725s were similar to the known Taxol biosynthesis hydroxylases. Interestingly, all these six CYP725 genes were expressed at the highest level in *T. Media*, and the lowest in *T. chinensis*. Their expression rule were coincident with Taxol content in *Taxus* organs. Therefore, they are likely to involved in Taxol biosynthesis, and their specific functions require further study.

### Cis-regulatory elements in the promoters of TCCYP725s

**Figure 8:**
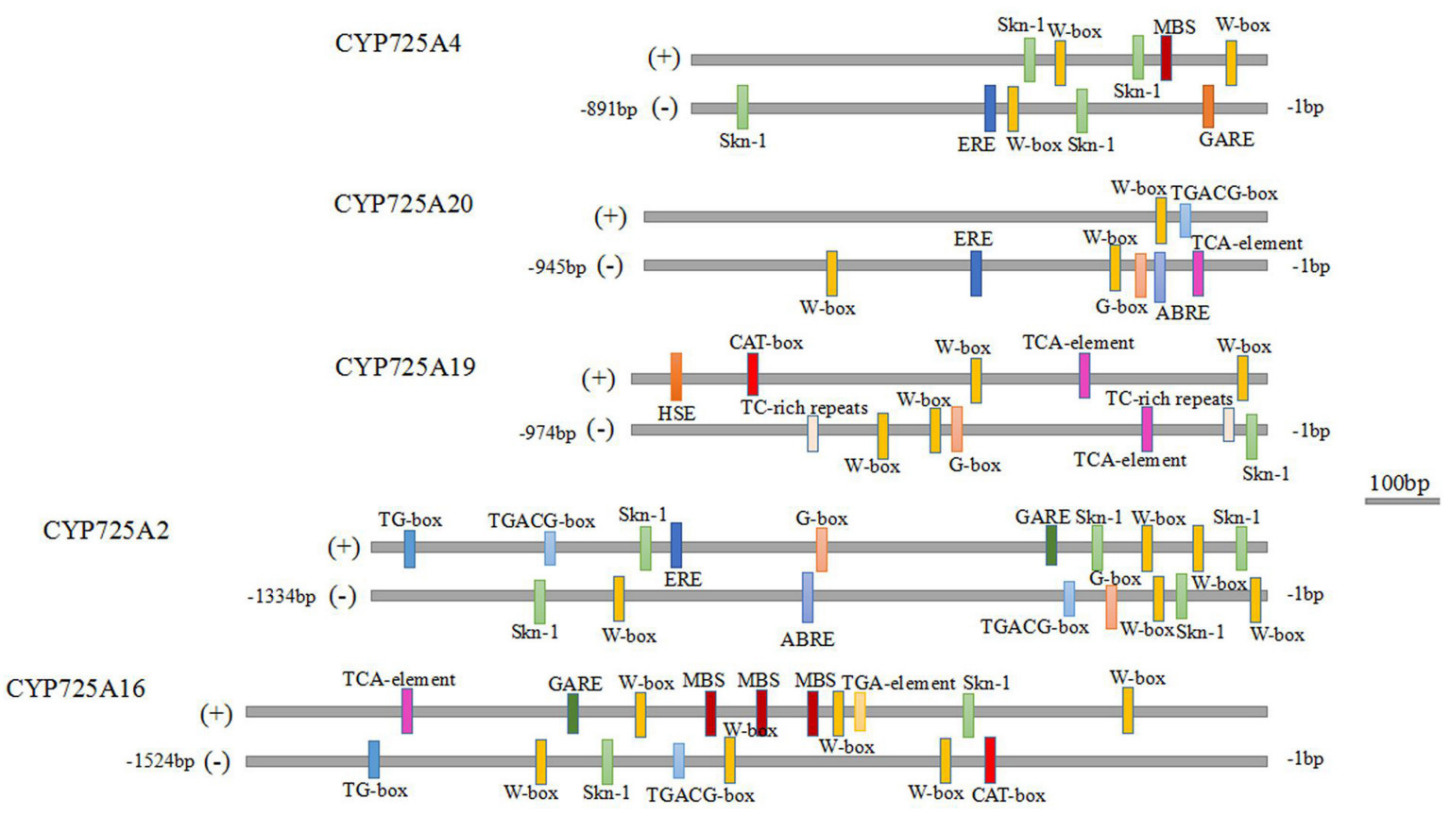
Cis-acting elements in the promoter regions of CYP725 genes. The scale bar represents 100 bp.

As *Taxus* genome information was not complete, only the promoter fragments of 5 TcCYP725s were identified (Accession Numbers: MF598831-MF598835). In addition to the common cis elements CAAT-box and TATA-box, 14 types of cis-acting elements in the TcCYP725s were discovered (Figure 8). The Skn-1 motif contributed to gene expression in the endosperm, and the CAT-box was required for gene expression in the meristem. The common cis-acting elements G-box and TG-box were responsive to light. All the other cis-regulatory elements are related to stresses and hormones, such as TC-rich repeats (required for defense and stresses), the TCA element (salicylic acid response), MBS (drought inducibility), HSE (heat response), W-box (responsive to fungal elicitors and plant hormones), the GARE motif (gibberellin response), the TGACG motif (MeJA response), ABRE (abscisic acid response), TGA (auxin response), and ERE (ethylene response). The Skn-1 motif is present in most of CYP725 genes, indicating that CYP725s play important roles in immature tissues. In addition, the W-box was widely found in CYP725 genes. Previous study showed that TcWRKY1 protein regulated Taxol biosynthesis in *T. chinensis* cells by specifically interacting with the two W-box of 10-deacetylbaccatin III-10β-O-acetyl transferase (DBAT; Li S. et al., 2013). These putative cis-acting elements would contribute to future researches of the transcriptional regulation of Taxol biosynthesis.

## Discussion

Taxol, a complex diterpenoid, is a highly effective antimitotic drug with excellent activity against many types of cancer. The lack of detailed information on *Taxus* CYP450s, such as centralized resource, systematic nomenclature, and biological functions, has significantly hampered research efforts to elucidate biosynthetic pathways for the medicinal ingredients in *Taxus*. To our knowledge, this study was the first to overcome these limitations by (1) identifying a large set of CYP450s; (2) establishing a systematic nomenclature for these CYP450s; (3) mining the candidate CYP450s that are likely related to Taxol biosynthesis; and (4) analyzing the cis-regulatory elements in the promoters of CYP725 genes to provides useful information to the transcriptional regulation of Taxol biosynthesis.

### *T. chinensis* CYP450s identified in this study

Totally, 118 full-length and 175 partial CYP450 genes were identified in *T. chinensis*. The number of CYP450 genes in *T. chinensis* is of the same order of magnitude as the number of CYP450s found in other gymnosperm and angiosperm plants, that is, 307 in *P. glauca*, 272 in *A. thaliana*, 332 in soybean, 334 in flax, and 455 in rice (Guttikonda et al., 2010; Babu et al., 2013; Warren et al., 2015). The previously identified five genes involved in Taxol biosynthesis, such as TcCYP725A1 (T10βH, AAN52360.1), TcCYP725A2 (T13αH, AAX59903.1), TcCYP725A4 (T5αH, AAU93341.1), TcCYP725A5 (T7βH, AAR21106.1), and TcCYP725A6 (T2αH, AAV54171.1), were included in the 118 full-length CYP450s. Moreover, TcCYP725A3, which is highly homologous with T14βH (Accession No. Q84KI1) in *T. cuspidata*, was explored in this study (Jennewein et al., 2003). This inferred that the CYP450s data in our research were representatives, and the remaining 112 novel full-length CYP450 sequences were of great value for the advanced research and applications of CYP450s in *Taxus*.

### Gymnosperm-specific CYP450s in *T. chinensis*

Some known gymnosperm-specific CYP450 subfamilies were discovered in *T. chinensis*. In the CYP71 clan, 8 CYP76AA and 18 CYP750 gymnosperm-specific members were identified among the 118 full-length CYP450s. Previous research revealed that the gymnosperms CYP76AA25 and CYP750B1 can catalyze the hydroxylation of sabinene to trans-sabin-3-ol from *Thuja plicata* (Bohlmann et al., 2015). The CYP85 clan, which includes many gymnosperm CYP450s, is involved in the biosynthesis of plant metabolites (Hamberger and Bohlmann, 2006; Zerbe et al., 2013): the CYP720B is a conifer-specific subfamily with four members in *T. chinensis*, and the CYP720B4 has been characterized in the biosynthesis of dehydroabietic acid, an ingredient related to the insect resistance of *Picea sitchensis* (Hamberger et al., 2011); the CYP716B is a gymnosperm-specific subfamily, with three members found in *T. chinensis*, and the sole functionally determined CYP716B gene is a taxoid 9α-hydroxylase in *Ginkgo biloba* (Zhang et al., 2014). Furthermore, *two* new gymnosperm-specific families were discovered in this study, namely, CYP864 in the 72 clan and CYP947 in the 85 clan (Communicated with Professor David Nelson, unpublished work. The gymnosperm CYP450 names have just been expanded by naming transcriptome data from the 1KP project). Two new gymnosperm-specific families that were recently discovered in *P. glauca*, including 71 clan family CYP867 and 72 clan family CYP866, were also found in *T. chinensis* (Warren et al., 2015). The exact functions of these new gymnosperm-specific families require further investigation.

### Potential candidate CYP450s involved in terpenoid biosynthesis

Terpenoids are one of the most widespread classes of secondary metabolites in higher plants, which are biosynthesized from basic isoprene units (C_5_H_8_) and further modified by various oxidoreductases, acyltransferases, dehydrogenases, and glucosyltransferases. CYP450-dependent oxidative modification is essential for the terpenoid biosynthesis. Hitherto, more than 50 CYP450 genes, which belong to CYP51, CYP71, CYP72, CYP76, CYP88, CYP93, CYP97, CYP701, CYP705, CYP706, CYP707, CYP714, CYP716, CYP720, CYP725, CYP735, and some unassigned families related to the biosynthesis of terpenoids in medicinal plants have been identified (Zhao et al., 2014). The structural diversity of terpenoid compounds depends on the rearrangement modifications of their skeletal structures and extensive oxidative modification (Zerbe et al., 2013). Therefore, it shouldn't be surprising that so many CYP450s families have been found related to their biosynthesis.

*Taxus spp*. specifically employs up to eight CYP450-mediated oxidatives to create the diterpene, Taxol (Jennewein and Croteau, 2001; Kaspera and Croteau, 2006). So far, the C-2, C-5, C-7, C-10, and C-13 hydroxylases have been successfully obtained (Jennewein et al., 2001, 2004; Schoendorf et al., 2001; Chau and Croteau, 2004; Chau et al., 2004). Unfortunately, the genes responsible for C-1 hydroxylation, oxetane formation, C-9 oxidation, and C-2′ hydroxylation remain unknown. In this study, an intriguing result was that 6 CYP725s (TcCYP725A9, TcCYP725A11, TcCYP725A16, TcCYP725A20, TcCYP725A22, and TcCYP725A23) were found to belong to the candidates that were involved in Taxol biosynthesis. By a blast search, TcCYP725A9, TcCYP725A11, TcCYP725A16, TcCYP725A22, and TcCYP725A23 were found to show high sequence similarity (>66%) to the T10βH (Schoendorf et al., 2001). The T10βH transformed taxadien-5α-yl acetate to taxadien-5α-acetoxy-10β-ol, which was an important intermediate in the biosynthesis of Taxol. In addition, the BLAST analysis of TcCYP725A20 revealed that the most homology (63%) found in public databases was with T13αH, an enzyme capable of hydrolyzing the taxadien-5α-ol at its C-13 position. The above information may also suggested that these 6 CYP725 genes were likely candidate contribute to the formation of Taxol.

### Future plans

Because the whole genome information of *T. chinensis* is unavailable, 5′ Race and 3′ Race can amplify numerous full-length CYP450 genes based on the current study. Functional predictions of the candidate CYP725s will be performed by heterologous expression in yeast. Linking *in vivo* feeding studies to cell-free enzyme systems, with available taxanes or suspension-cultured cells as ingredients, will enable better understanding of the Taxol biosynthetic pathway. Ultimately, we can reconstruct this secondary metabolite pathway in a microbial system to establish the engineered production of Taxol. Moreover, the further researches of the transcriptional regulation of Taxol biosynthesis will be done, based on the putative cis-acting elements in the promoters of taxoid hydroxylase.

## Author contributions

Conceived and designed the experiments: CF, LY, WL. Performed the experiments: WL, SZ, KD. Analyzed the data: MZ, KD. Contributed reagents/materials/analysis tools: SZ, YC. Wrote the paper: WL.

### Conflict of interest statement

The authors declare that the research was conducted in the absence of any commercial or financial relationships that could be construed as a potential conflict of interest.
