# Supplementary material for: Transcriptome Assembly and Systematic Identification of Novel Cytochrome P450s in Taxus chinensis
Source: Front Plant Sci. 2017 Aug 23;8:1468. doi: 10.3389/fpls.2017.01468 (PMC5572210; doi:10.3389/fpls.2017.01468)
Supplement: Supplementary file 1 [file DataSheet1.DOC]

**Supplementary information**

**Figure legends**

**Figure S1 Conserved motifs of 118 full-length CYP450s from *T. chinensis*.** The names of the motifs are indicated above each logo.

**Figure S2 KEGG pathway analyses of predicted CYP450 genes in *T. chinensis*.** The numbers of CYP450 genes involved in the corresponding metabolic processes are shown.

**Figure S1**


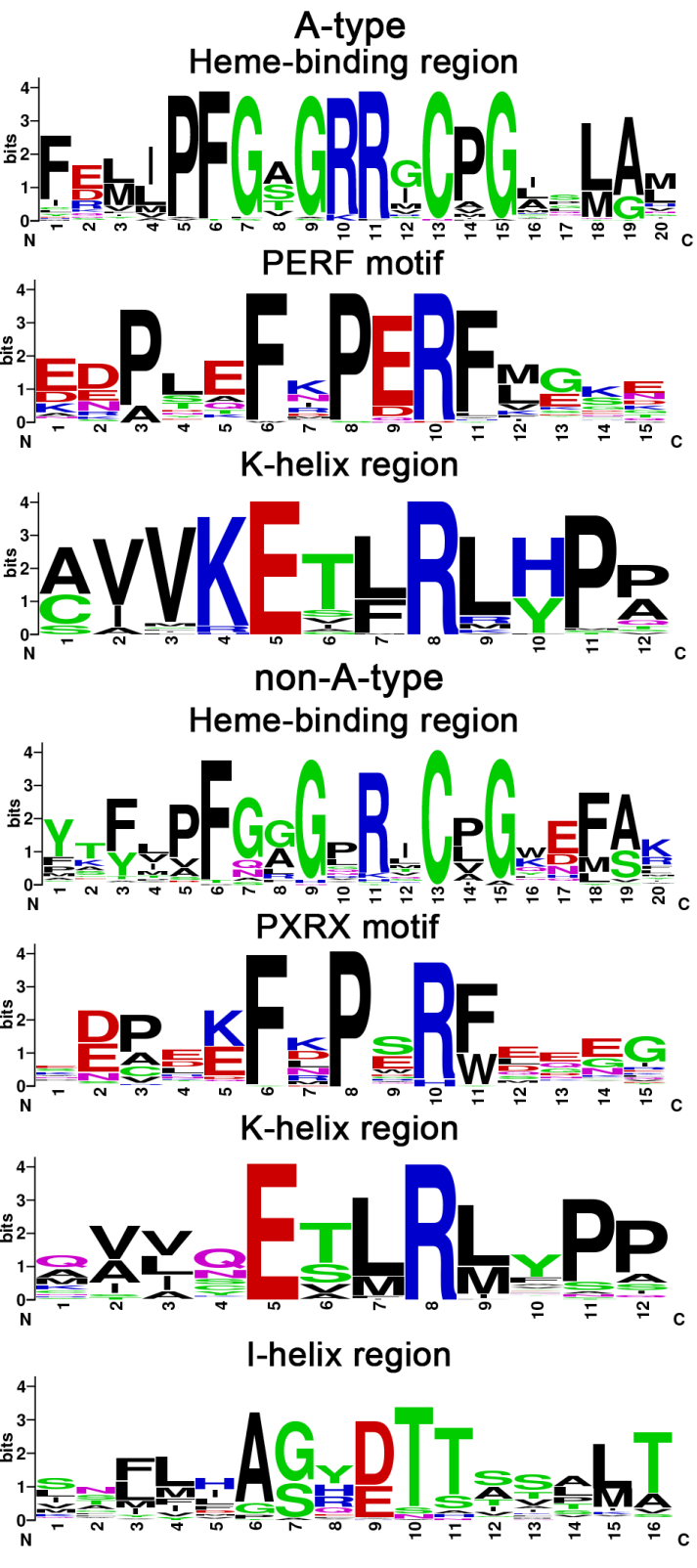


**Figure S2**

**
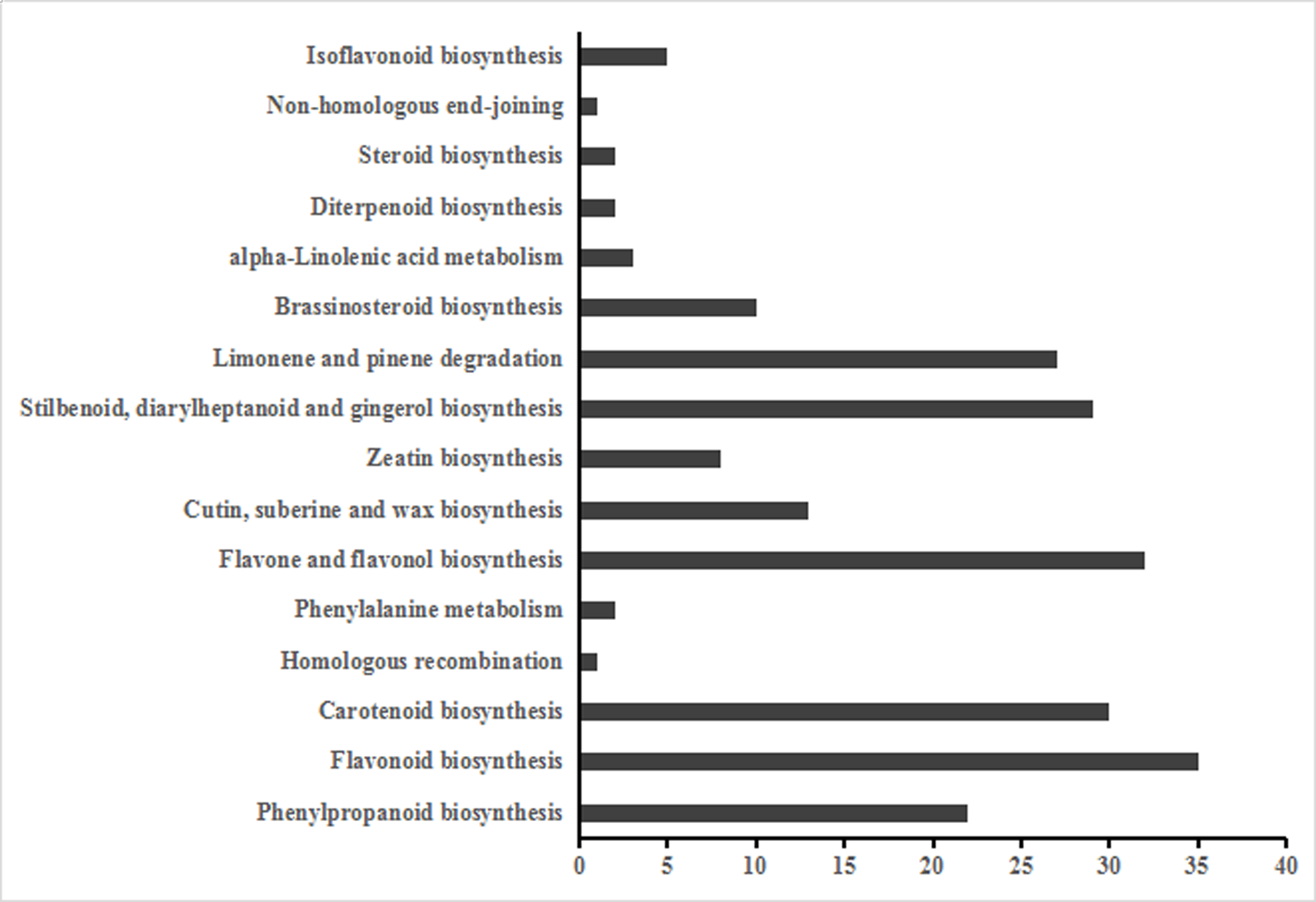
**

**Supplementary Information**

**Table S1. Primers used for qRT-PCR.**

| Gene name | Forward Primer (5’-3’) | Reverse Primer (5’-3’) |
| --- | --- | --- |
| TcCYP73A170 | TGCCAGTGCTGTGGAAGATA | GGGCCTGAGAATAGGAATAA |
| TcCYP75A77 | CAAAGTGGTTGGTCGTGATAGG | TCAGAAGAGACTCGTGGTAGGC |
| TcCYP75B115 | AAAGCGAGCGAGTTTAGGGAG | CAAATCGAGCCAAGCCAAGGA |
| TcCYP76AA72 | TTTGGGGTGAATGTGGAG | TGGTGCGAAATACTTGGT |
| TcCYP94P6 | ATCGTGGGTTCCCTGTTTT | GTTCGCTGGATTTGCTGTT |
| TcCYP716B29 | TTGCTATCGCCTGTGACTTGT | ATTTGGCTTTGCGGTACCTTG |
| TcCYP716B30 | GGGTTGGGAAGGAAGAAGT | CCGAGGCTGACAAAAAGAG |
| TcCYP718B1 | GAAAGCAAGGAACCAGGAGA | GATTGGAGGCAACAACCGCA |
| TcCYP725A3 | GAAGGGCAACGACCAAGT | CCGACGGTAAGCAAATCC |
| TcCYP725A9 | ATCTTGCGTTCGGCTCT | CATCTTTTCCCTTCCAT |
| TcCYP725A10 | CTCAAGCGTTGCAGAATTATATACC | TGCAATGGAGAAGATTAGGCCTC |
| TcCYP725A11 | AAATTCAAGCCTTCGAGAT | GGAATAAGTTTCATGCGTTG |
| TcCYP725A12 | TTCGATGAGCCCGAGAAATTTAG | TTCCCATCCTGCACATATGCGTAC |
| TcCYP725A13 | TGCTTCCTTTGATGAAGGGCCTCG | ATAGTTCCCACACCAACAGTTT |
| TcCYP725A14 | AAAGGAAGGGGAAGAAATCA | ACTGGTGGGTACATTCGTAG |
| TcCYP725A15 | TCTACTTTCGGTGTTGCTCA | CTTCTCCGTCCTTTTTATTC |
| TcCYP725A16 | TCCAATATACTTCACGCCTC | TGATTTCTTCTCCGTCCTTC |
| TcCYP725A17 | TGGCCTTGATATTTAAGCTTCTA | AAATTGCAGGAAACATCCTTAAT |
| TcCYP725A18 | GACGAGGAAGAAAAGGAACGA | GTGAGCAACACGGAGACCAG |
| TcCYP725A19 | GGCAACGACCAAGTGAGTGTA | GCCCGACGGTAAGCAAAT |
| TcCYP725A20 | CTCATGGGCGAGGACTCTG | TGGTCCGAAGAAGGTGTCAA |
| TcCYP725A21 | ATCACCGCCATTGTTGCCAC | ATGAACCGAATCGACTCCCC |
| TcCYP725A22 | AGGAAGGCAGGCATGTGG | GGGGTCAACTGGGATGTAGC |
| TcCYP725A23 | CGTCATAGAAACCGATCCTCTG | CGAAGTCTTGAAAACACCACCA |
| TcCYP728Q11 | TTTCTTCCATACTTCCTCTCC | TTGTTACACTTCCGTGCCCTC |
| TcCYP728Q12 | GAGGGATGAAAACGGCAATG | GCCAACAGACGAACAAGATG |
| TcCYP728S3 | AAGAGAGAAATAGCAGAGGGA | GAACAGCAGTGGTATCGTGAC |
| TcCYP750C3 | CCCACAGAGAAAGAATGC | CAAGGTCACGAAGAGCAC |
| TcCYP750C24 | AGGAGGGCTTTGGATGA | TTGGCTACACGGGGGTT |
| TcCYP750C29 | AGGGTTTTGTGGATGTGCT | TCGGTAACTATGCGATGGT |
| Actin | GGTATCCACGAGACGA | ACCCTCCAATCCAGAC |

**Table S2. Expression levels (FPKM) of full-length CYP450 genes from two *T. chinensis* cell lines: CA and NA.**

|  | Expression levels (FPKM) | |  |
| --- | --- | --- | --- |
| Gene Name | CA | NA | FDR |
| TcCYP51G1 | 17.4427 | 9.8969 | 2.41E-159 |
| TcCYP73A170 | 4.1836 | 932.0704 | 1.52E-79 |
| TcCYP73A171 | 0.3022 | 64.9015 | 4.21E-75 |
| TcCYP74A73 | 121.7386 | 103.8301 | 2.30E-139 |
| TcCYP74A74 | 0.203 | 18.7782 | 1.94E-96 |
| TcCYP74A75 | 0.5106 | 4.7743 | 1.04E-51 |
| TcCYP75A77 | 0.4844 | 452.2046 | 1.04E-51 |
| TcCYP75B115 | 9.0432 | 678.235 | 7.66E-43 |
| TcCYP76AA66 | 223.12 | 52.5579 | 3.79E-30 |
| TcCYP76AA67 | 0 | 14.7271 | 3.21E-58 |
| TcCYP76AA68 | 29.7609 | 4.5208 | 3.53E-69 |
| TcCYP76AA69 | 1154.0355 | 294.9529 | 2.90E-29 |
| TcCYP76AA70 | 34.1784 | 0.9815 | 1.12E-28 |
| TcCYP76AA71 | 0.1822 | 16.8006 | 2.30E-186 |
| TcCYP76AA72 | 44.6247 | 179.1919 | 5.01E-89 |
| TcCYP76Z4 | 0.0287 | 21.0543 | 4.21E-75 |
| TcCYP77F31 | 369.6873 | 192.7735 | 1.69E-27 |
| TcCYP77F32 | 88.277 | 62.7521 | 3.11E-160 |
| TcCYP77F34 | 1.2463 | 0.0613 | 2.53E-26 |
| TcCYP77F35 | 1.6999 | 16.4079 | 4.35E-28 |
| TcCYP782B7 | 27.5337 | 23.0382 | 3.90E-80 |
| TcCYP78A233 | 1.6343 | 0.992 | 5.69E-24 |
| TcCYP78A234 | 14.0499 | 2.3676 | 8.08E-38 |
| TcCYP86A149 | 1.827 | 0.0212 | 1.48E-103 |
| TcCYP86J5 | 2.0383 | 6.1985 | 4.56E-41 |
| TcCYP86P14 | 0.7011 | 1.0351 | 1.59E-37 |
| TcCYP90A54 | 0 | 0.112 | 2.21E-23 |
| TcCYP94D79 | 4.3543 | 4.2255 | 4.20E-126 |
| TcCYP94D80 | 7.0367 | 6.0711 | 2.17E-121 |
| TcCYP94D81 | 4.1214 | 3.1552 | 1.69E-27 |
| TcCYP94D83 | 3.6212 | 3.179 | 3.31E-45 |
| TcCYP94P4 | 33.5662 | 23.7888 | 3.76E-20 |
| TcCYP94P5 | 3.5158 | 1.1273 | 3.57E-23 |
| TcCYP94P6 | 0.0595 | 8.6687 | 2.93E-55 |
| TcCYP97A57 | 14.6142 | 11.337 | 3.79E-30 |
| TcCYP701A59 | 9.5541 | 9.5643 | 1.24E-105 |
| TcCYP703D4 | 1.0059 | 0.0966 | 8.48E-118 |
| TcCYP704C10 | 3.331 | 0.2321 | 2.16E-18 |
| TcCYP710A78 | 45.6289 | 18.9159 | 1.95E-72 |
| TcCYP715D11 | 45.338 | 30.1243 | 1.78E-40 |
| TcCYP715C54 | 16.6589 | 1.4446 | 2.42E-36 |
| TcCYP716B29 | 11.7138 | 12.2236 | 1.68E-22 |
| TcCYP716B30 | 25.6127 | 4.0516 | 7.48E-56 |
| TcCYP718B1 | 13.0167 | 64.4822 | 6.51E-27 |
| TcCYP720B23 | 8.5314 | 2.5929 | 3.85E-64 |
| TcCYP720B24 | 3.4262 | 1.0445 | 2.10E-59 |
| TcCYP720B25 | 71.4548 | 37.2256 | 2.90E-29 |
| TcCYP725A1 | 16.0623 | 30.4599 | 1.13E-205 |
| TcCYP725A2 | 6.5798 | 37.4345 | 2.01E-161 |
| TcCYP725A3 | 22.6944 | 140.1793 | 1.51E-42 |
| TcCYP725A4 | 11.8434 | 13.6346 | 2.31E-41 |
| TcCYP725A5 | 0.8374 | 276.356 | 5.32E-39 |
| TcCYP725A6 | 0.6208 | 13.3889 | 1.59E-37 |
| TcCYP725A9 | 76.9398 | 172.2891 | 1.26E-97 |
| TcCYP725A10 | 5.2358 | 7.3101 | 1.43E-34 |
| TcCYP725A11 | 6.6546 | 19.3331 | 1.64E-66 |
| TcCYP725A12 | 13.0701 | 5.1166 | 1.92E-25 |
| TcCYP725A13 | 11.6979 | 5.0506 | 3.36E-158 |
| TcCYP725A14 | 4.8533 | 1.2358 | 6.18E-50 |
| TcCYP725A15 | 1.1013 | 1.1866 | 2.23E-156 |
| TcCYP725A16 | 3.3856 | 9.0405 | 2.88E-157 |
| TcCYP725A17 | 1.4354 | 1.445 | 1.64E-17 |
| TcCYP725A18 | 6.1525 | 66.8405 | 1.64E-17 |
| TcCYP725A19 | 0.7802 | 12.23 | 2.69E-157 |
| TcCYP725A20 | 4.173 | 27.2581 | 3.41E-36 |
| TcCYP725A21 | 65 | 93.8449 | 2.23E-156 |
| TcCYP725A22 | 8.5053 | 28.9027 | 1.78E-40 |
| TcCYP725A23 | 0.3908 | 62.022 | 2.13E-91 |
| TcCYP728Q11 | 30.1301 | 2.0914 | 6.91E-40 |
| TcCYP728Q12 | 0.0642 | 11.255 | 2.99E-42 |
| TcCYP728S2 | 999.1734 | 208.7699 | 8.50E-11 |
| TcCYP728S3 | 20.0604 | 98.4077 | 2.23E-11 |
| TcCYP729B25 | 2.9312 | 1.3682 | 8.34E-18 |
| TcCYP736E20 | 10.1671 | 3.9676 | 1.12E-23 |
| TcCYP736E21 | 9.5801 | 26.6214 | 2.98E-12 |
| TcCYP736E22 | 5.0551 | 118.7929 | 8.49E-11 |
| TcCYP750B2 | 0 | 12.8967 | 1.47E-24 |
| TcCYP750C18 | 0.5957 | 0.088 | 6.31E-17 |
| TcCYP750C19 | 0.1554 | 0.2142 | 1.68E-22 |
| TcCYP750C20 | 1.6142 | 30.7168 | 4.35E-11 |
| TcCYP750C21 | 7.7696 | 0.6264 | 1.22E-36 |
| TcCYP750C22 | 0.2812 | 0.9227 | 3.28E-32 |
| TcCYP750C23 | 7.9251 | 6.403 | 2.49E-11 |
| TcCYP750C24 | 5.1142 | 0.4815 | 1.14E-11 |
| TcCYP750C25 | 3.4546 | 13.0901 | 6.51E-27 |
| TcCYP750C8 | 35.7916 | 105.1952 | 2.89E-24 |
| TcCYP750C3 | 65.9818 | 77.3465 | 6.51E-22 |
| TcCYP750C26 | 409.9735 | 386.7675 | 3.23E-10 |
| TcCYP750C27 | 0.2259 | 2.5844 | 3.31E-27 |
| TcCYP750C28 | 22.4934 | 16.9372 | 2.97E-12 |
| TcCYP750C29 | 2.134 | 5.0532 | 2.71E-14 |
| TcCYP864B7 | 128.1369 | 21.4399 | 2.16E-18 |
| TcCYP866A17 | 62.4239 | 102.9551 | 3.76E-20 |
| TcCYP866A18 | 7.5255 | 4.9466 | 2.05E-51 |
| TcCYP866A19 | 8.4981 | 6.378 | 1.04E-13 |
| TcCYP866A20 | 14.8168 | 4.2617 | 2.33E-18 |
| TcCYP866B7 | 41.955 | 39.0853 | 3.43E-33 |
| TcCYP867B5 | 36.0977 | 15.3273 | 1.83E-15 |
| TcCYP867F22 | 73.3899 | 26.1068 | 3.04E-15 |
| TcCYP867F23 | 65.6662 | 128.0426 | 2.71E-14 |
| TcCYP947A88 | 166.331 | 141.3607 | 3.21E-17 |

**Table S3. Expression levels (FPKM) of the identified transcripts involved in the Taxol biosynthetic pathway.**

|  | Expression levels (FPKM) | |  |
| --- | --- | --- | --- |
| Gene Name | CA | NA | FDR |
| AACT | 43.5774 | 12.4442 | 5.11E-150 |
| HMGS | 18.2524 | 2.2509 | 2.22E-74 |
| HMGR | 27.5268 | 7.2035 | 2.98E-13 |
| MK | 10.4606 | 5.2041 | 1.27E-06 |
| PMK | 13.0294 | 6.4828 | 2.00E-09 |
| MDC | 12.5057 | 6.223 | 5.05E-15 |
| IDI | 5.7687 | 2.8709 | 1.27E-06 |
| DXS | 0.5247 | 5.5338 | 8.59E-15 |
| DXR | 1.2844 | 13.5621 | 3.38E-45 |
| MECT | 17.9336 | 8.9614 | 4.43E-30 |
| CMK | 1.6628 | 17.2116 | 1.12E-28 |
| MECPS | 367.547 | 696.7537 | 1.02E-05 |
| HDS | 252.8561 | 438.0269 | 2.32E-46 |
| HDR | 169.5056 | 265.4854 | 7.22E-15 |
| GPPS | 13.1707 | 65.436 | 1.69E-27 |
| FPPS | 665.2156 | 933.4539 | 3.11E-160 |
| GGPPS | 9.1891 | 17.5625 | 2.53E-26 |
| TS | 0.5785 | 17.8685 | 4.35E-28 |
| T2αH | 0.6208 | 13.3889 | 1.59E-37 |
| T5αH | 11.8434 | 13.6346 | 2.30E-14 |
| T7βH | 0.8374 | 276.356 | 5.32E-39 |
| T13αH | 6.5798 | 37.4345 | 1.86E-60 |
| TDAT | 11.6842 | 64.0323 | 1.46E-21 |
| T10βH | 16.0623 | 30.4599 | 2.02E-11 |
| T14βH | 22.6944 | 140.1793 | 1.51E-42 |
| DBT | 0.7011 | 2.3384 | 1.30E-62 |
| DBAT | 2.6926 | 0.6523 | 3.00E-51 |
| BAPT | 2.3513 | 4.3265 | 4.20E-126 |
| DBTNBT | 1.0367 | 3.0721 | 2.17E-121 |

**Table S4. Expression levels (FPKM) of full-length *T. chinensis* CYP450 genes under MeJA elicitationin.**

|  | Expression levels (FPKM) | |  |
| --- | --- | --- | --- |
| Gene Name | Tm0 | Tm16 | FDR |
| TcCYP51G1 | 45.0281 | 11.948 | 3.45E-11 |
| TcCYP73A170 | 113.5432 | 41.2675 | 2.81E-06 |
| TcCYP73A171 | 42.3344 | 16.1446 | 5.27E-52 |
| TcCYP74A73 | 98.6132 | 96.6886 | 2.42E-29 |
| TcCYP74A74 | 440.1218 | 1847.9764 | 1.05E-06 |
| TcCYP75A77 | 13.205 | 7.5972 | 1.05E-06 |
| TcCYP75B115 | 25.1182 | 23.9826 | 2.39E-60 |
| TcCYP76AA66 | 150.5084 | 204.8269 | 6.72E-13 |
| TcCYP76AA67 | 25.961 | 52.5381 | 2.51E-13 |
| TcCYP76AA68 | 19.4059 | 19.491 | 3.92E-07 |
| TcCYP76AA69 | 547.4494 | 25.7559 | 3.92E-07 |
| TcCYP76AA70 | 3.5622 | 6.8296 | 3.92E-07 |
| TcCYP76AA71 | 25.961 | 52.5381 | 5.99E-20 |
| TcCYP76AA72 | 19.4059 | 19.491 | 5.75E-39 |
| TcCYP76AA73 | 36.7548 | 32.7723 | 3.86E-237 |
| TcCYP76Z4 | 5.432 | 4.9706 | 5.45E-107 |
| TcCYP77F32 | 11.6038 | 6.3588 | 1.31E-14 |
| TcCYP77F33 | 15.3783 | 6.8631 | 6.09E-237 |
| TcCYP782B7 | 52.3658 | 24.8691 | 6.80E-16 |
| TcCYP78A233 | 2.087 | 1.1485 | 2.04E-08 |
| TcCYP78A234 | 1.7221 | 0.5832 | 6.26E-63 |
| TcCYP86J5 | 61.0834 | 80.423 | 1.80E-40 |
| TcCYP90A54 | 2.9848 | 6.1189 | 7.62E-09 |
| TcCYP94D79 | 23.0493 | 30.5757 | 2.84E-09 |
| TcCYP94D81 | 72.3006 | 32.9962 | 1.06E-09 |
| TcCYP94D83 | 23.8265 | 17.5343 | 1.06E-09 |
| TcCYP94P4 | 23.606 | 58.6002 | 3.96E-10 |
| TcCYP94P5 | 28.9604 | 114.9612 | 5.30E-70 |
| TcCYP94P6 | 0 | 1.2547 | 5.55E-11 |
| TcCYP94P7 | 28.0503 | 110.562 | 1.92E-25 |
| TcCYP97A57 | 27.6189 | 17.0686 | 5.10E-225 |
| TcCYP97B52 | 6.0993 | 4.6041 | 1.02E-277 |
| TcCYP701A59 | 23.4324 | 15.6658 | 5.62E-14 |
| TcCYP703D4 | 2.7337 | 1.2258 | 2.99E-42 |
| TcCYP704C10 | 3.2084 | 4.4943 | 3.03E-55 |
| TcCYP710A78 | 19.9272 | 343.2126 | 1.46E-169 |
| TcCYP715D11 | 114.3098 | 85.9365 | 1.04E-34 |
| TcCYP715C54 | 0.5279 | 0 | 6.59E-111 |
| TcCYP716B29 | 27.3609 | 27.4305 | 6.09E-29 |
| TcCYP716B30 | 7.887 | 27.313 | 1.10E-69 |
| TcCYP718B1 | 45.4561 | 106.2021 | 3.78E-12 |
| TcCYP720B23 | 11.4758 | 11.0868 | 4.76E-49 |
| TcCYP720B24 | 84.5034 | 75.8611 | 3.31E-45 |
| TcCYP720B26 | 115.9815 | 135.5968 | 3.31E-45 |
| TcCYP725A1 | 1.2889 | 22.6354 | 4.24E-18 |
| TcCYP725A2 | 7.4494 | 8.4546 | 6.48E-50 |
| TcCYP725A3 | 1.7854 | 1.8059 | 3.39E-54 |
| TcCYP725A4 | 2.3604 | 16.2127 | 1.64E-17 |
| TcCYP725A5 | 0.4963 | 6.7533 | 3.37E-10 |
| TcCYP725A6 | 17.2825 | 27.2296 | 1.39E-18 |
| TcCYP725A9 | 172.2981 | 417.2016 | 1.97E-23 |
| TcCYP725A10 | 2.3377 | 2.0594 | 1.27E-14 |
| TcCYP725A11 | 168.9025 | 243.391 | 2.16E-104 |
| TcCYP725A12 | 4.7199 | 14.1301 | 1.68E-06 |
| TcCYP725A13 | 3.6853 | 13.791 | 6.78E-22 |
| TcCYP725A14 | 10.3213 | 6.6266 | 2.57E-25 |
| TcCYP725A16 | 13.3371 | 37.9871 | 3.37E-10 |
| TcCYP725A19 | 5.1037 | 2.1065 | 2.37E-10 |
| TcCYP725A20 | 6.4442 | 19.4608 | 1.70E-19 |
| TcCYP725A21 | 24.9005 | 18.4966 | 2.16E-29 |
| TcCYP725A22 | 17.9472 | 35.5196 | 3.22E-05 |
| TcCYP725A23 | 0.4251 | 7.6026 | 2.77E-46 |
| TcCYP728Q11 | 26.2825 | 11.7184 | 1.76E-15 |
| TcCYP728Q12 | 3.437 | 0.7308 | 7.57E-52 |
| TcCYP728Q13 | 0.895 | 8.2194 | 1.39E-07 |
| TcCYP728S2 | 1111.2002 | 129.0696 | 2.46E-16 |
| TcCYP728S3 | 225.5445 | 33.5957 | 2.36E-07 |
| TcCYP729B25 | 12.498 | 11.7588 | 4.79E-18 |
| TcCYP736C8 | 9.857 | 2.6971 | 1.83E-05 |
| TcCYP736E20 | 5.6781 | 2.1535 | 4.49E-06 |
| TcCYP736E21 | 13.6664 | 32.2486 | 8.60E-05 |
| TcCYP736E23 | 5.6781 | 2.1535 | 1.96E-16 |
| TcCYP736E24 | 0.4698 | 2.327 | 2.56E-17 |
| TcCYP750B2 | 2.1451 | 2.6334 | 1.47E-34 |
| TcCYP750C16 | 97.9473 | 61.2499 | 3.22E-05 |
| TcCYP750C17 | 18.7862 | 1.6663 | 8.91E-11 |
| TcCYP750C18 | 188.2524 | 94.6803 | 2.81E-06 |
| TcCYP750C19 | 4.8256 | 2.0307 | 3.33E-11 |
| TcCYP750C20 | 7.1051 | 5.5668 | 3.28E-08 |
| TcCYP750C21 | 97.9473 | 61.2499 | 2.31E-07 |
| TcCYP750C22 | 101.3247 | 49.047 | 3.82E-174 |
| TcCYP750C23 | 136.1567 | 1.374 | 3.07E-133 |
| TcCYP750C24 | 545.6649 | 72.3153 | 1.81E-05 |
| TcCYP750C25 | 5.2445 | 15.1688 | 6.27E-50 |
| TcCYP750C8 | 4.4871 | 2.4884 | 8.59E-45 |
| TcCYP750C3 | 297.3496 | 70.7029 | 3.13E-36 |
| TcCYP750C27 | 51.2647 | 42.5144 | 3.69E-41 |
| TcCYP750C28 | 10.1796 | 6.5609 | 3.87E-41 |
| TcCYP750C30 | 170.3688 | 43.9771 | 2.09E-20 |
| TcCYP864B7 | 47.2649 | 83.9066 | 1.50E-19 |
| TcCYP866A17 | 121.7115 | 165.4904 | 1.47E-16 |
| TcCYP866A18 | 24.1546 | 34.7887 | 2.83E-15 |
| TcCYP866A19 | 119.6446 | 150.3628 | 2.89E-56 |
| TcCYP866A20 | 51.8876 | 15.0287 | 2.18E-38 |
| TcCYP866B7 | 62.8348 | 84.9538 | 5.85E-38 |
| TcCYP866B10 | 10.8014 | 0.9808 | 3.45E-12 |
| TcCYP867B5 | 63.3867 | 381.2092 | 2.15E-23 |
| TcCYP867E3 | 164.6882 | 777.7412 | 2.74E-107 |
| TcCYP867F22 | 385.9473 | 503.1553 | 5.34E-11 |
| TcCYP867F23 | 136.1168 | 192.452 | 5.34E-11 |
| TcCYP867G20 | 4.4355 | 21.7463 | 1.02E-09 |

**Table S5. qRT-PCR confirmation of the expression profiles of some randomly selected CYP450 genes.**

|  | Relative expression | |
| --- | --- | --- |
| Gene Name | CA | NA |
| TcCYP725A9 | 1 | 1.426 |
| TcCYP725A16 | 1 | 1.507 |
| TcCYP725A3 | 1 | 4.235 |
| TcCYP750C29 | 1 | 1.396 |
| TcCYP728S3 | 1 | 1.933 |
| TcCYP728Q11 | 1 | 0.105 |
| TcCYP75B115 | 1 | 6.232 |
| TcCYP73A170 | 1 | 1.958 |
| TcCYP716B29 | 1 | 9.893 |
| TcCYP716B30 | 1 | 0.516 |
| TcCYP750C24 | 1 | 36.511 |
| TcCYP750C3 | 1 | 21.801 |
| TcCYP718B1 | 1 | 24.633 |
| TcCYP76AA72 | 1 | 32.687 |
| TcCYP75A77 | 1 | 15.136 |
| TcCYP94P6 | 1 | 62.365 |
| TcCYP728Q12 | 1 | 17.812 |
